# Cardioprotective Effect of Nanoencapsulated α-Amyrin in Spontaneously Hypertensive Rats with Metabolic Syndrome

**DOI:** 10.3390/ph19101599

**Published:** 2026-10-09

**Authors:** Gabriela Pereira da Silva, Luana Ale Bertoncello Pael, Maria Luiza Fidelis da Silva, Maria Medina de Azevedo, Telma Lélia Gonçalves Schultz de Carvalho, Annye Vitória Moraes, Harveli Darpy Balouta, Leticia de Andrade Maldonado Aires, Tatiane Pereira de Souza, Serafim Florentino Neto, Maria de Fatima da Silva Bezerra, Jesús Rafael Rodríguez Amado, Emerson Luiz Botelho Lourenço, Arquimedes Gasparotto Junior

**Affiliations:** 1 Laboratory of Cardiovascular Pharmacology (LaFaC), Faculty of Health Sciences, Federal University of Grande Dourados (UFGD), Dourados 79804-970, MS, Brazil; gabriela.pereirasilva01@gmail.com (G.P.d.S.); mariamedinaazevedo@outlook.com (M.M.d.A.);; 2 Laboratory of Innovation and Development in Pharmaceutical Technology (LIDTEF), Faculty of Pharmaceutical Sciences, Federal University of Amazonas (UFAM), Manaus 69080-900, AM, Brazil; 3 Graduate Program in Biotechnology Applied to Agriculture, Paranaense University, Umuarama 87502-210, PR, Brazil

**Keywords:** cardioprotection, hypertension, polymeric nanocapsules, *Protium* spp.

## Abstract

**Background/Objectives:** Metabolic syndrome (MS) encompasses a cluster of disorders that significantly increase cardiovascular risk. While α-amyrin exhibits antioxidant and anti-inflammatory properties, its poor aqueous solubility and low oral bioavailability hinder its clinical use. This study evaluated the cardioprotective effects of α-amyrin-loaded polymeric nanocapsules (NCαA) in spontaneously hypertensive rats (SHR) with sucrose-induced MS. **Methods:** First, NCαA was prepared via interfacial deposition of a preformed polymer following organic solvent displacement. The formulation was characterized for particle size, zeta potential, morphology, encapsulation efficiency, and storage stability. Spontaneously hypertensive rats (SHR) received 20% sucrose for 8 weeks and were treated daily by oral gavage for 28 days with NCαA (0.1, 0.3, or 1 mg/kg), unformulated α-amyrin (1 mg/kg), hydrochlorothiazide (25 mg/kg), vehicle, or blank nanocapsules. Cardiovascular, electrocardiographic, echocardiographic, vascular, renal, and biochemical parameters were subsequently assessed. **Results:** Polymeric NCαA showed spherical and uniform morphology (195.10 ± 1.55 nm; PDI: 0.144; zeta potential: −52.60 mV), 85.85% encapsulation efficiency, efficient sustained release, and 90-day shelf-life stability. In vivo, MS control rats exhibited significant weight gain, hypertension, increased S-wave negative deflection depth, reduced ejection fraction, impaired acetylcholine-mediated vasodilation, depressed serum nitrite, and elevated malondialdehyde. Treatment with NCαA (particularly at 0.3 and 1 mg/kg), unlike unformulated α-amyrin, prevented excessive weight gain, normalized blood pressure, reversed electrophysiological/echocardiographic markers of cardiac overload, restored nitric oxide bioavailability, attenuated lipid peroxidation, and maintained renal hydroelectrolytic balance. **Conclusions:** Polymeric nanoencapsulation yielded stable nanocapsules with high sustained release, overcoming the biopharmaceutical limitations of α-amyrin. NCαA provides comprehensive cardioprotection against MS-induced damage, representing a promising multi-target strategy for cardiometabolic disorders.

## 1. Introduction

Metabolic syndrome (MS) is a multifactorial clinical condition characterized by a cluster of metabolic disturbances that increase the risk of cardiovascular disease, type 2 diabetes mellitus, and other comorbidities [1,2]. According to the criteria established by the International Diabetes Federation (IDF) [3], the diagnosis of MS requires the presence of central obesity associated with at least two of the following factors: arterial hypertension, elevated fasting glucose, hypertriglyceridemia, or reduced HDL cholesterol. This combination significantly contributes to increased cardiovascular morbidity and mortality. Epidemiological data underscore the magnitude of this challenge; the World Health Organization estimated that approximately 890 million adults lived with obesity in 2022, while the IDF reported approximately 589 million adults with diabetes worldwide in 2024 [3,4]. This scenario highlights the urgent need for effective therapeutic strategies for the management of MS and its associated cardiovascular complications.

The treatment of MS generally requires a multifactorial approach, often involving polypharmacy and lifestyle modifications, which can hinder therapeutic adherence and increase the risk of adverse effects [5]. In this context, new pharmacological strategies are being investigated, including the use of nanostructured systems capable of improving the bioavailability, therapeutic efficacy, and safety profiles of existing drugs. Studies have demonstrated that nanoformulations of compounds such as metformin and statins exhibit superior therapeutic efficacy with a lower incidence of adverse reactions compared to conventional formulations [6,7,8].

**Figure 1:**
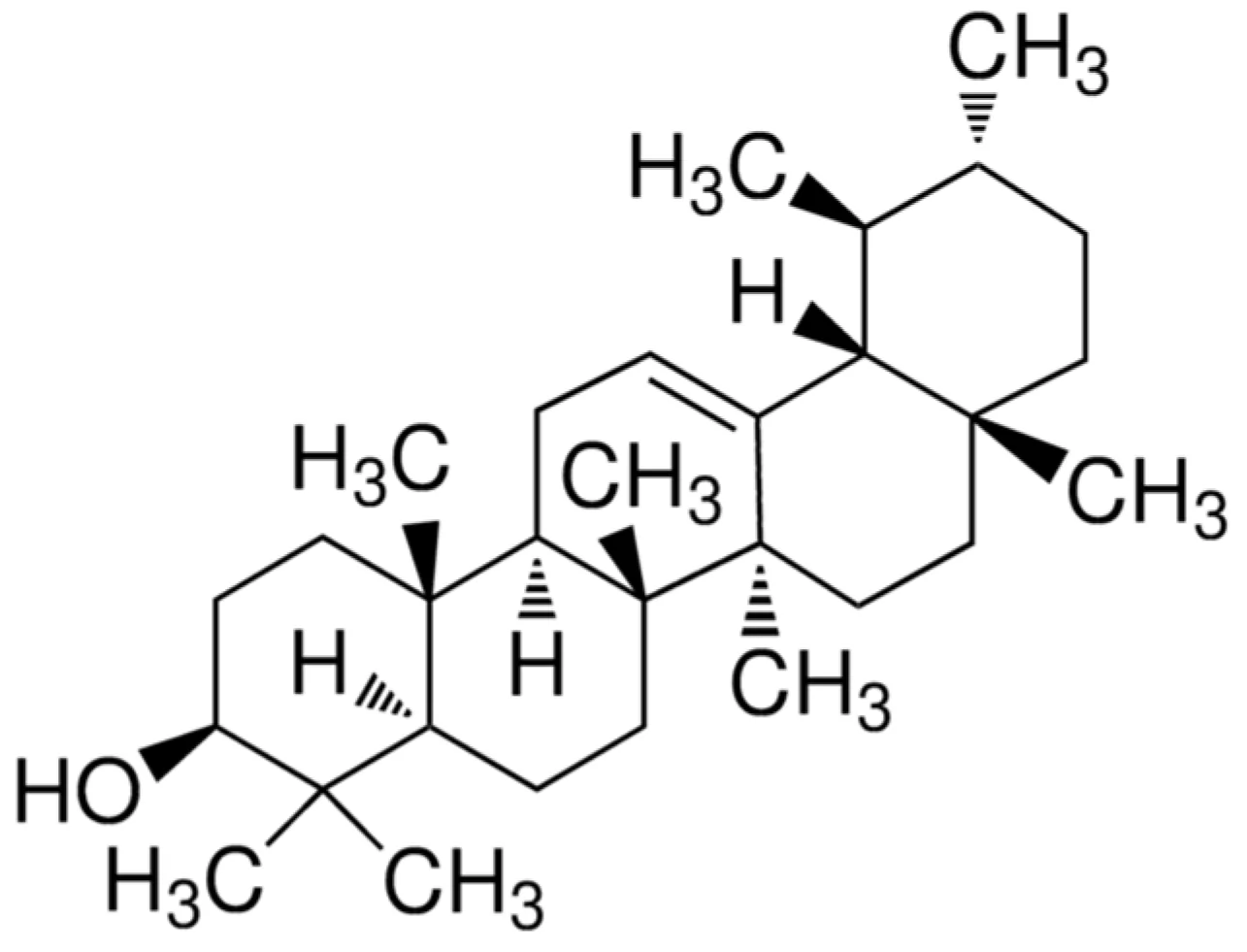
Molecular structure of α-amyrin.

Alpha-amyrin (αA) is a pentacyclic ursane-type triterpenoid containing a double bond between positions 12 and 13, with a hydroxy group at the beta-position (Figure 1). Species of the genus *Protium* Burm.f. (Burseraceae), popularly known as ‘breu-branco,’ are rich in αA, which is well-documented for its anti-inflammatory, antioxidant, and hypolipidemic properties [9,10,11]. Despite its therapeutic potential, the clinical application of αA is hindered by its low water solubility and reduced oral bioavailability—limitations that may be overcome through nanoencapsulation in polymeric systems [12].

The field of nanoparticles is constantly expanding, playing a pivotal role in diverse biomedical applications such as bioimaging, diagnostics, and targeted drug administration. Within pharmaceutical development, nanoencapsulation using biocompatible and biodegradable polymers offers a promising strategy to overcome the physicochemical limitations of therapeutic compounds. Among various nanocarriers, nanocapsules have attracted significant attention due to their distinctive core–shell architecture—typically classified into polymeric nanocapsules, consisting of a core surrounded by a polymeric membrane, and lipid nanocapsules, which feature an oily core stabilized by surfactant shells. These systems enhance drug stability, accommodate high loading capacities, and enable controlled release kinetics while minimizing burst release [12,13]. In particular, pH-responsive acrylic polymers provide enteric solubility characteristics by remaining insoluble in acidic gastric fluids and dissolving selectively at neutral-to-alkaline intestinal pH [14,15].

Therefore, to address the poor solubility and stability constraints of αA while enhancing its intestinal delivery, this study focused on the development and physicochemical characterization of αA-loaded polymeric nanocapsules (NCαA), followed by the investigation of their cardioprotective efficacy and metabolic impact in an experimental model of metabolic syndrome induced in spontaneously hypertensive rats (SHR).

## 2. Results

### 2.1. Synthesis and Characterization of NcαA

#### 2.1.1. Preparation of NCαA

**Figure 2:**
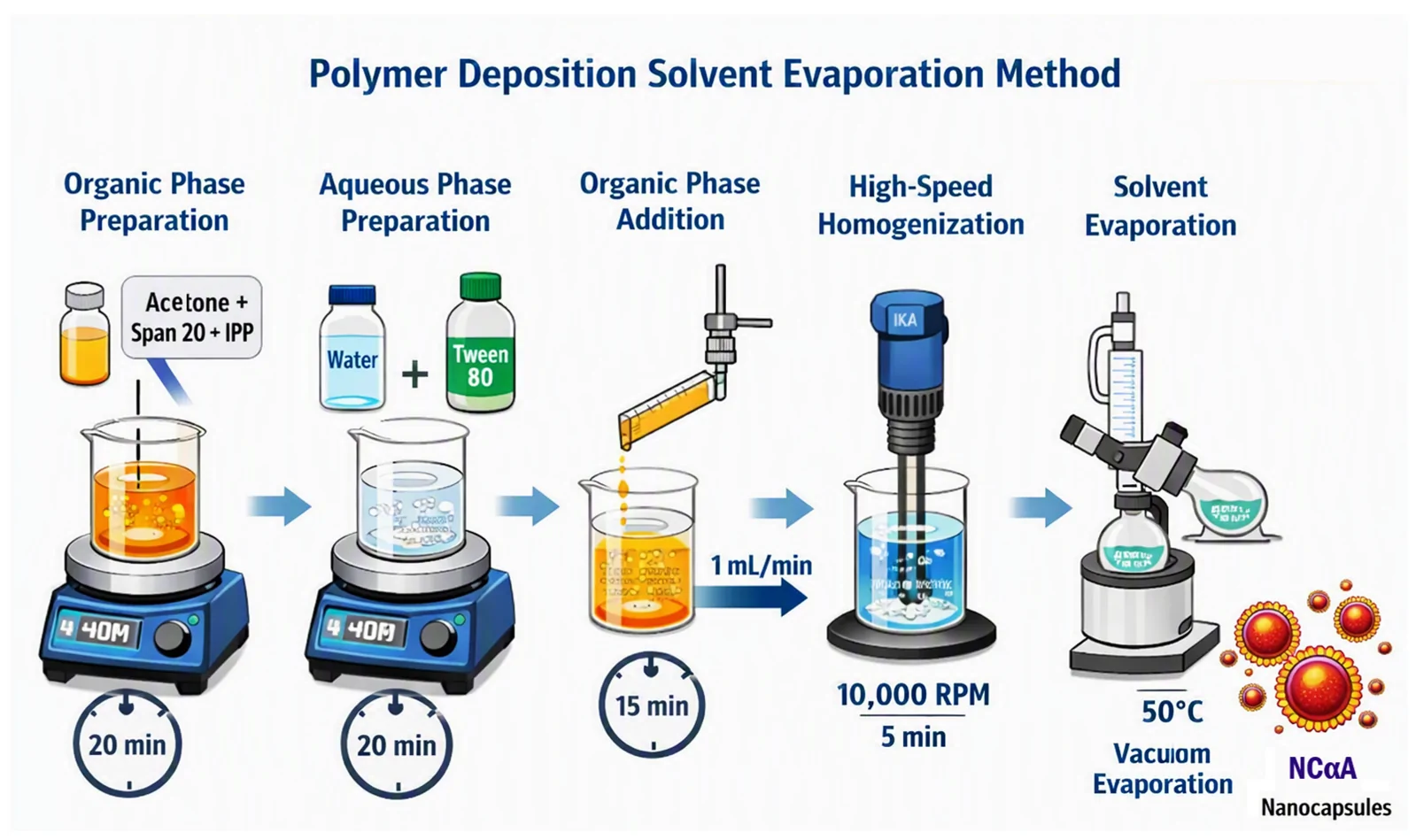
Schematic diagram of the preparation of α-amyrin-loaded polymeric nanocapsules (NCαA). IPP, isopropyl palmitate.

As shown in Figure 2, nanocapsules were successfully prepared via interfacial deposition of a preformed polymer following organic solvent displacement. Briefly, the organic phase—containing the polymer (forming the polymeric wall), isopropyl palmitate (acting as the oily core), and sorbitan monolaurate (Span^®^ 20, Croda International Plc, Goole, UK), serving as the lipophilic surfactant—dissolved in ethanol (organic solvent) was prepared under magnetic stirring at 400 rpm. Simultaneously, the aqueous phase, composed of distilled water (aqueous medium) and polysorbate 80 (Tween^®^ 80, Croda International Plc, Goole, UK) (acting as the hydrophilic surfactant), was prepared under identical conditions. The organic phase was then added to the aqueous phase under controlled infusion, and the resulting mixture was subjected to high-shear homogenization at 10,000 rpm for 5 min using an Ultra-Turrax homogenizer (IKA, Staufen, Germany). Finally, the organic solvent was removed by rotary evaporation at 50 °C under reduced pressure, yielding the final nanocapsule formulation.

#### 2.1.2. NCαA Size, Morphology, and Zeta Potential

**Figure 3:**
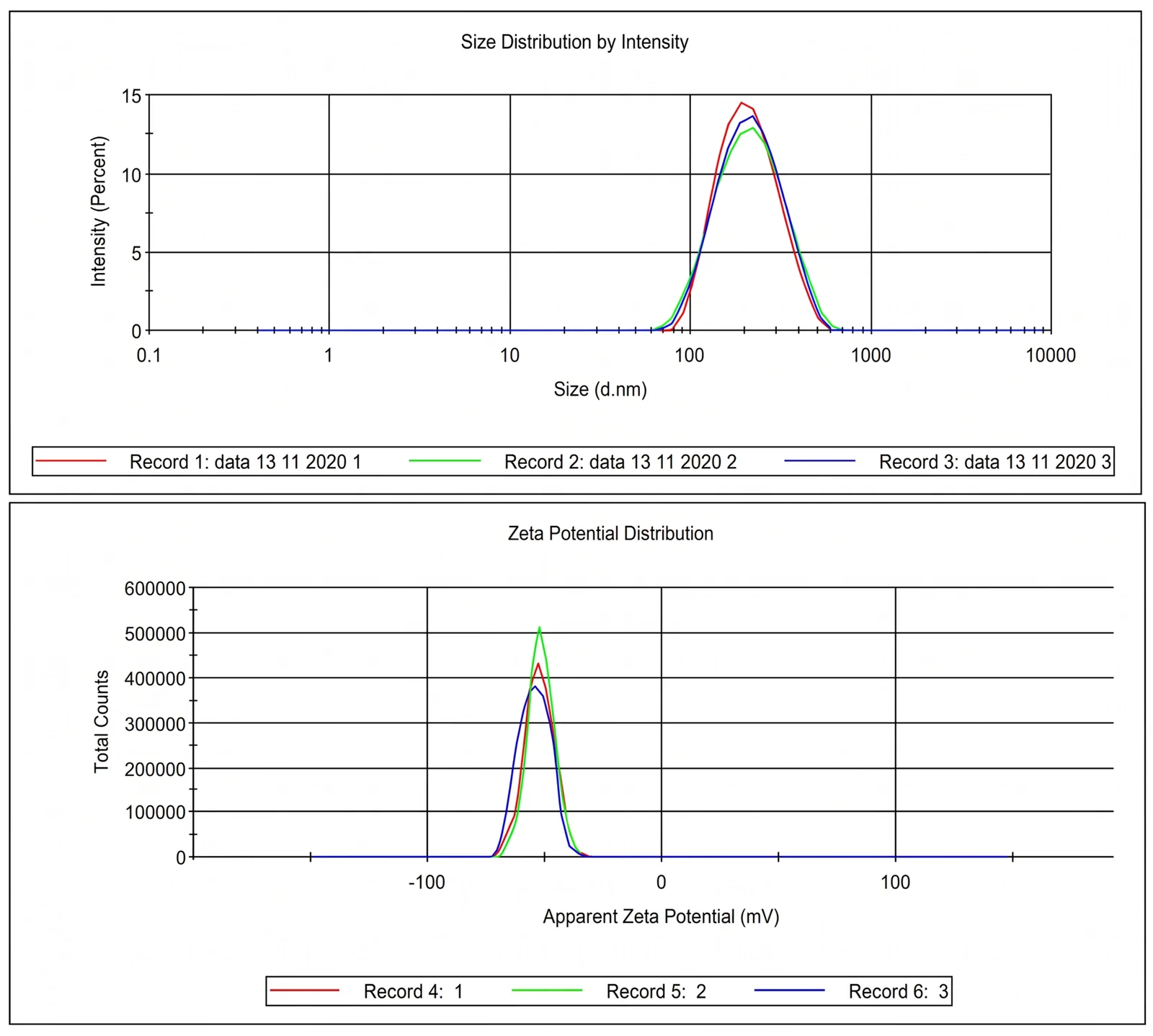
Particle size distribution and zeta potential of α-amyrin-loaded nanocapsules (NcαA).

The NCαA exhibited an average hydrodynamic diameter of 195.10 ± 1.55 nm with a polydispersity index (PDI) of 0.144 ± 0.005. The zeta potential, determined by electrophoretic light scattering (ELS), was −52.60 ± 1.25 mV (Figure 3). The particle size distribution curves displayed a single, well-defined peak centered around 190 nm, indicating the formation of nanocapsules within the expected nanoscale range. The three measurements exhibited high overlap, demonstrating excellent reproducibility. The low PDI value confirms a homogeneous population with minimal size variability. Furthermore, the zeta potential distribution showed a distinct peak near −50 mV with consistent profiles across replicates, indicating robust surface charge characteristics that contribute to colloidal stability.

**Figure 4:**
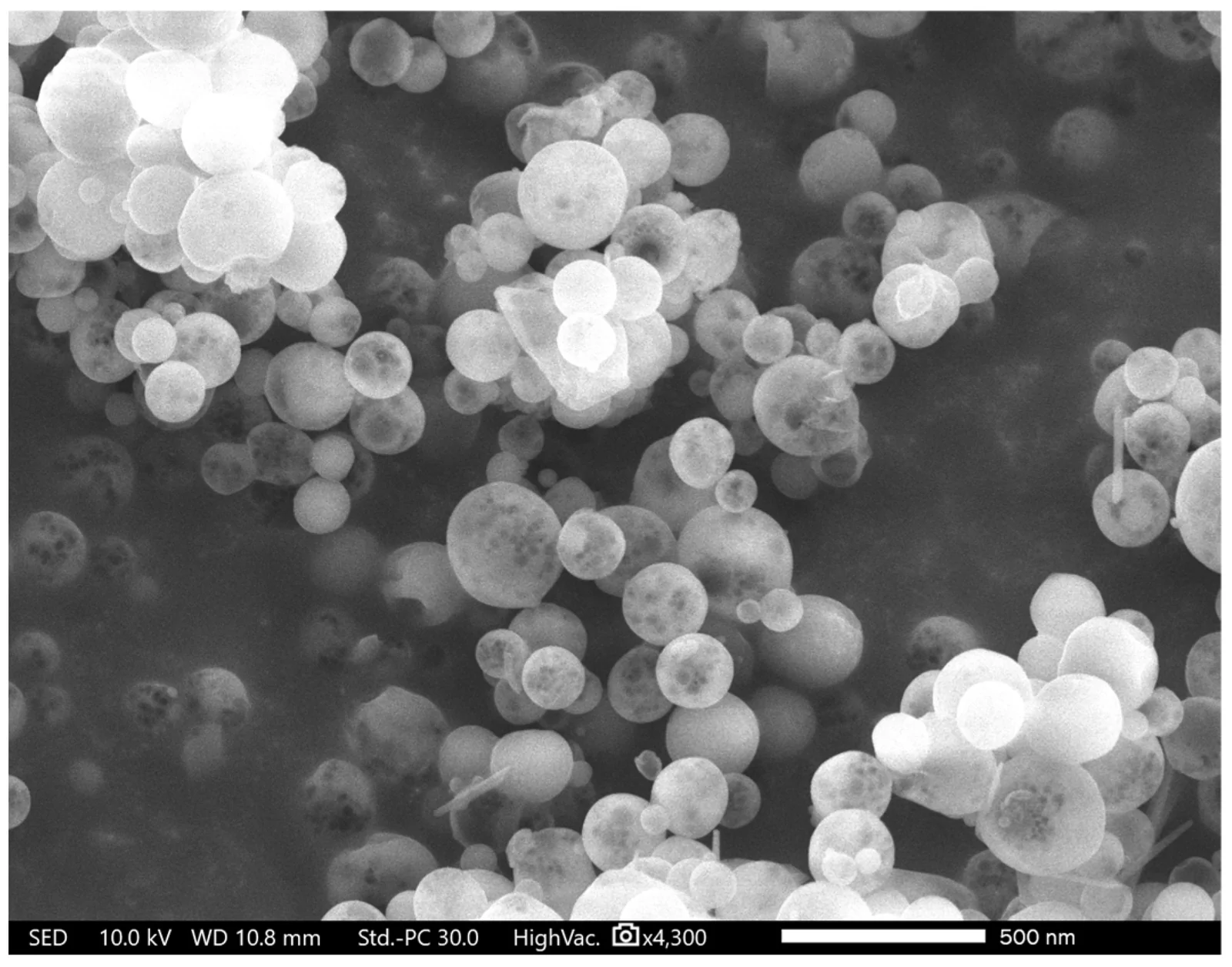
Scanning electron microscopy (SEM) micrograph of α-amyrin-loaded nanocapsules (NcαA).

Scanning electron microscopy (SEM) micrographs revealed the spherical morphology of the NCαA, characterized by smooth and uniform surfaces (Figure 4). The particles appeared densely packed without visible structural defects. High-vacuum imaging conditions (10.0 kV, working distance of 10.8 mm) enabled clear visualization of their morphology, confirming the successful formation of well-defined nanocapsule walls.

#### 2.1.3. Release Profile

**Figure 5:**
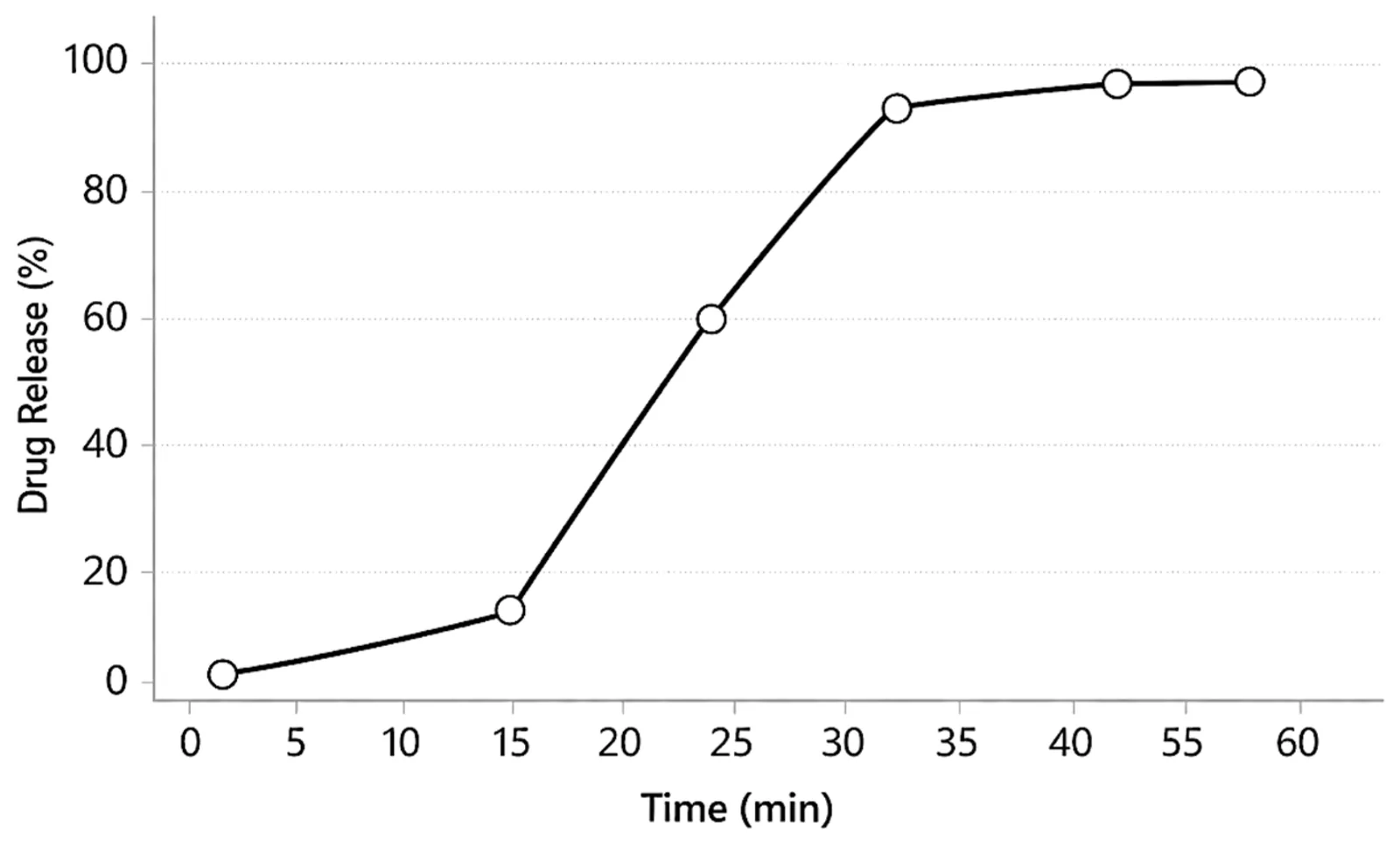
In vitro release profile of α-amyrin-loaded nanocapsules (NcαA).

Figure 5 shows the in vitro release profile of NCαA. The profile exhibited an initial slow phase with approximately 13% of drug release within the first 15 min, followed by a rapid release phase reaching 60% at 25 min and 93% at 32 min. A plateau was achieved between 42 and 58 min, with maximum cumulative release approaching 97%.

#### 2.1.4. Encapsulation Efficiency

The NCαA exhibited an encapsulation efficiency of 85.85 ± 2.23%, indicating that a high proportion of the compound was successfully incorporated into the nanocapsules.

#### 2.1.5. Shelf-Life Stability Study

Throughout the 90-day stability evaluation, the nanocapsules maintained a consistent particle size of approximately 195 nm, with minimal variation across time points (Table 1). The polydispersity index remained low (0.144–0.147), indicating a homogeneous size distribution throughout storage. The zeta potential remained strongly negative, ranging from −52.60 mV to −56.65 mV, with no significant fluctuations that would suggest instability.

### 2.2. In Vivo Pharmacological Assays

#### 2.2.1. Renal Function

Throughout the experimental period, no behavioral changes, alterations in capillary glycemia, or clinical signs of toxicity were observed in any of the experimental groups. Table 2 presents the effects of HCTZ (hydrochlorothiazide), αA (α-amyrin), and NCαA on 24 h urinary volume and renal electrolyte excretion across the different experimental groups. On the first day of treatment, only the HCTZ-treated group showed a significant increase in urinary volume and renal excretion of sodium, potassium, and chloride. By day 28, a significant reduction in urinary volume and renal electrolyte excretion was observed in the NC (negative control), Blank (blank polymeric nanocapsule group; Table S1), NCαA (0.1 mg/kg), and αA (1 mg/kg) groups compared with naïve rats. However, treatment with HCTZ or NCαA (0.3 and 1 mg/kg) prevented these changes, maintaining values comparable to those of the naïve group.

#### 2.2.2. Body Weight Gain and Electrocardiographic Parameters

Table 3 presents the body weight gain (final weight − initial weight) and the electrocardiographic profile of the experimental groups treated with vehicle, HCTZ (positive control), αA (1 mg/kg), or different doses (0.1, 0.3, and 1 mg/kg) of NCαA. All rats in the NC and Blank (Table S1) groups showed an increase in body weight gain compared to animals in the naïve group (which did not receive sucrose). Animals treated with the lowest dose of NCαA, as well as αA, showed body weight gain similar to that observed in the NC and Blank groups. No statistical differences in body weight gain were observed among the other experimental groups when compared to naïve rats. Regarding the electrocardiogram, SHRs in the NC, Blank, NCαA (0.1 mg/kg), and αA (1 mg/kg) groups exhibited a significant increase in the absolute depth of the S-wave negative deflection compared with the naïve group. In contrast, treatment with HCTZ or NCαA (0.3 and 1 mg/kg) prevented these changes, maintaining the S-wave negative deflection at levels similar to those of the naïve group. No statistically significant differences were observed in the other electrocardiographic parameters evaluated.

#### 2.2.3. Transthoracic Echocardiography

End-diastolic volume (EDV), end-systolic volume (ESV), ejection fraction (EF), left ventricular end-diastolic diameter (LVEDD), left ventricular end-systolic diameter (LVESD), and stroke volume (SV) across the experimental groups (naïve, NC, HCTZ, αA, and NCαA) are presented in Table 4. Animals in the NC, Blank (Table S1), αA (1 mg/kg), and NCαA (0.1 mg/kg) groups exhibited a significant reduction in EF, as well as a significant increase in LVESD compared to normotensive animals (naïve group). Prolonged treatment (28 days) with HCTZ and NCαA (0.3 and 1 mg/kg) normalized these echocardiographic parameters compared with the NC and Blank groups, maintaining EF and LVESD values comparable to those of the naïve group. No statistically significant differences in EDV, ESV, LVEDD, and SV were observed among the experimental groups.

#### 2.2.4. Blood Pressure and Heart Rate (HR)

Systolic blood pressure (SBP), diastolic blood pressure (DBP), mean arterial pressure (MAP), and heart rate (HR) data for the different experimental groups (naïve, NC, HCTZ, αA, and NCαA) are shown in Table 5. Animals in the NC, Blank (Table S1), αA (1 mg/kg), and NCαA (0.1 mg/kg) groups showed a significant elevation in SBP, DBP, and MAP compared with normotensive animals (naïve group). Prolonged treatment (28 days) with HCTZ and NCαA (0.3 and 1 mg/kg) significantly reduced blood pressure parameters compared with the NC and Blank groups, maintaining SBP, DBP, and MAP values comparable to those of the naïve group. No statistically significant differences in HR were observed among the studied groups.

#### 2.2.5. Reactivity of Resistance Arteries

The reactivity of the mesenteric vascular bed (MVB) to phenylephrine (Phe), acetylcholine (ACh), and sodium nitroprusside (SNP) in the different experimental groups is presented in Table 6. Compared to normotensive controls, the NC, Blank (Table S1), NCαA (0.1 mg/kg), and αA (1 mg/kg) groups exhibited significantly increased Phe-induced vasoconstriction (10 and 30 nmol) and impaired ACh-induced vasodilation (30 nmol). Prolonged treatment with HCTZ or NCαA (0.3 and 1 mg/kg) prevented these changes, maintaining vascular reactivity at levels comparable to those observed in naïve rats.

#### 2.2.6. Serum Biochemical Parameters

The serum biochemical parameters for the different experimental groups are presented in Table 7. Hypertensive-SM rats in the NC, Blank (Table S1), HCTZ, and αA (1 mg/kg) groups exhibited a significant reduction in serum nitrite levels alongside increased malondialdehyde (MDA) levels compared to the naïve group. Prolonged treatment with NCαA at doses of 0.3 and 1 mg/kg prevented these alterations, maintaining nitrite and MDA levels similar to those observed in naïve rats. Furthermore, higher doses of NCαA (0.3 and 1 mg/kg) elevated serum nitrotyrosine (NT) levels to values significantly higher than those observed in the NC, Blank, HCTZ, NCαA (0.1 mg/kg), αA (1 mg/kg), or naïve groups. In contrast, triglycerides and serum insulin levels were significantly elevated in all hypertensive-SM rats, regardless of the treatment administered. None of the other evaluated parameters were significantly altered across the experimental groups.

#### 2.2.7. Relative Target Organ Weight

The effects of NCαA on relative target organ weights on day 29 are presented in Table 8. Regardless of the treatment, all hypertensive-MS rats exhibited left ventricular hypertrophy, as indicated by a significant increase in relative heart weight compared to the naïve group. Regarding the left kidney, the NC, Blank (Table S1), and αA (1 mg/kg) groups showed a significant increase in relative weight compared to naïve rats; however, treatment with HCTZ and all doses of NCαA effectively attenuated this change, maintaining values similar to those of the naïve group. Furthermore, relative liver weight was significantly elevated in the NC, HCTZ, αA (1 mg/kg), and low-dose NCαA (0.1 and 0.3 mg/kg) groups compared to naïve animals. Conversely, the highest dose of NCαA (1 mg/kg) prevented this alteration, keeping relative liver weight comparable to that of the naïve group. No significant changes were observed in the relative weights of the spleen and right kidney across the experimental groups.

#### 2.2.8. Histopathological Analysis

The Supplementary Materials (Figure S1) present representative histological sections of the liver, left kidney, left ventricle, and aortic arch from the naïve, NC, Blank, HCTZ, NcαA (1 mg/kg), and αA (1 mg/kg) groups. No evidence of leukocyte infiltration, inflammation, fibrosis, hypoplasia, hyperplasia, hypertrophy, ischemia, apoptosis, or necrosis was detected in the analyzed tissues across any of the experimental groups. The semi-quantitative evaluation yielded a score of 0 for all examined organs, demonstrating the absence of lesions and the preservation of normal tissue architecture across all evaluated slides.

## 3. Discussion

Pentacyclic triterpenes, such as α-amyrin, possess well-documented anti-inflammatory, antioxidant, and hypolipidemic actions [9,10,11]. However, the clinical translation of these promising natural compounds is frequently hampered by poor biopharmaceutical properties, particularly high lipophilicity, low aqueous solubility, and erratic gastrointestinal absorption [16,17]. To overcome these long-standing formulation challenges, we developed polymeric nanocapsules featuring a core–shell architecture (NCαA).

Physicochemical characterization confirmed that NCαA exhibited a submicron hydrodynamic diameter with a unimodal, highly homogeneous size distribution, alongside a spherical morphology and smooth surfaces. These results align with previous reports from our research group, corroborating the feasibility and reproducibility of the preparation method [16]. Furthermore, these structural features were reinforced by a high encapsulation efficiency (85.85 ± 2.23%), reflecting the reliability of the manufacturing process.

Nonetheless, it is important to highlight that our methodology quantified total non-free α-amyrin in the supernatant following centrifugation, without physically isolating surface-adsorbed molecules from true core-entrapped drug fractions. Given the pronounced lipophilicity of α-amyrin (log *p* > 7) and the absence of a prominent initial in vitro burst release, the active compound is reasonably expected to partition preferentially into the oily isopropyl palmitate core. Nevertheless, direct quantification of surface adsorption remains an analytical limitation of this study, and future investigations will implement selective desorption techniques to precisely decouple core encapsulation from surface-associated fractions.

The nanocapsules showed excellent stability, evidenced by a strongly negative zeta potential (−52.60 ± 1.25 mV). According to the Derjaguin–Landau–Verwey–Overbeek (DLVO) theory [18], colloidal stability depends on the balance between attractive van der Waals forces, which draw particles together to induce aggregation, and repulsive forces arising from the overlapping electrical double layers of identically charged particles. In this system, the strongly negative zeta potential significantly amplifies electrostatic repulsion. When this repulsion substantially exceeds the attractive van der Waals forces, a high repulsive energy barrier develops along the net interaction energy profile. This barrier prevents the nanocapsules from approaching closely enough to fall into the deep primary energy minimum associated with irreversible coagulation, thereby maintaining colloidal integrity and preventing agglomeration throughout prolonged storage.

The in vitro release profile of the NCαA was evaluated in PBS buffer (pH 7.4) as the dissolution medium (Figure 5). The nanocapsules displayed a characteristic sigmoidal pattern governed by distinct kinetic stages. During the initial lag phase (0–15 min), only approximately 13% of the drug was released, reflecting the time required for medium penetration, wetting, and initial hydration of the polymeric shell surrounding the oily isopropyl palmitate/amyrin core. The absence of an immediate, massive burst confirms that the highly lipophilic active compound is effectively sequestered within the core rather than weakly adsorbed onto the particle surface. Following this induction period, an accelerated release phase occurred between 15 and 35 min, during which drug release rapidly escalated from 13% to 60% at 25 min and reached 93% by 32 min. This rapid efflux is driven by polymer relaxation, swelling, and potential shell erosion as the carboxylic groups of the methacrylic copolymer ionize, significantly lowering the diffusion barrier and permitting rapid outward mass transfer along the steep concentration gradient [19]. Between 40 and 60 min, the system entered an asymptotic plateau phase, achieving a maximum cumulative release of approximately 97%, which demonstrates near-complete drug payload availability under sink conditions with negligible irreversible entrapment within the residual polymer matrix. Mechanistically, this behavior indicates anomalous (non-Fickian) transport involving a coupled mechanism of macromolecular relaxation and concentration-driven diffusion [20]. Furthermore, the strong electrostatic repulsion of the colloidal system—evidenced by its negative zeta potential—prevents particle aggregation throughout the assay, ensuring an optimal and uniform specific surface area across the colloidal dispersion that facilitates synchronized and rapid drug release once the integrity of the polymeric barrier is altered [16].

To date, pharmaceutical nanotechnology platforms designed to encapsulate α-amyrin remain limited. The majority of reported approaches have utilized lipid-based nanoemulsions to circumvent the low aqueous solubility of triterpenes—either employing an α- and β-amyrin isomeric mixture [21] or surface-modified nanoemulsions for targeted routes, such as chitosan-decorated droplets for intranasal delivery [22]. Additionally, multiple studies have encapsulated crude botanical extracts or isomeric mixtures (e.g., from *Protium* or *Euphorbia* species) into liposomes, cyclodextrin complexes, or nanospheres. While Alsaedi et al. [23] explored the systemic biological effects of α-amyrin nanocapsules in diabetic models, comprehensive physicochemical profiling and polymer interface characterization were not fully explored. Consequently, building upon our group’s foundational work with methacrylic acid-based polymeric nanocapsules [16], the present formulation represents a distinct, thoroughly characterized core–shell polymeric system specifically engineered for purified α-amyrin, offering improved colloidal stability and controlled release properties compared to standard lipidic or crude isomeric delivery systems.

By improving gastrointestinal dispersion, preventing premature degradation, and extending systemic half-life [24,25,26], this nanostructured platform optimized drug bioavailability, effectively circumventing the low aqueous solubility and poor oral bioavailability that traditionally limit the therapeutic delivery of unformulated α-amyrin (αA) through its core–shell polymeric architecture. To validate these biopharmaceutical advantages, metabolic syndrome (MS) was induced in spontaneously hypertensive rats (SHRs) via prolonged 20% sucrose administration, providing a translational model that mirrors human metabolic and hemodynamic dysregulations [27] driven by fructose-fueled de novo lipogenesis [28,29,30]. While sucrose-control and blank groups exhibited pronounced weight gain, hypertriglyceridemia, hyperinsulinemia, systemic oxidative stress, endothelial dysfunction, hypertension, and left ventricular overload, prolonged treatment with nanoencapsulated αA (NCαA at 0.3 and 1 mg/kg) prevented most of these metabolic and cardiovascular alterations, corroborating evidence that αA prevents lipid disturbances and improves insulin sensitivity [10]. Strikingly, whereas NCαA demonstrated significant and multifaceted cardioprotection, unformulated αA at 1 mg/kg failed to elicit any significant response, thereby confirming the indispensable role of polymeric nanocapsules in mediating the systemic efficacy of this pentacyclic triterpene and highlighting nanoencapsulation as a pivotal strategy for therapeutic translation.

Beyond the metabolic improvements described above, the most prominent hemodynamic finding of this study was the significant effect of NCαA on blood pressure regulation. Untreated animals in the NC and Blank groups exhibited significant elevations in SBP, DBP, and MAP. In contrast, prolonged treatment with NCαA at doses of 0.3 and 1 mg/kg led to a significant reduction in all blood pressure parameters, successfully restoring them to values similar to those observed in the normotensive (naïve) group. On the other hand, the absence of significant changes in HR across all experimental groups indicates that this antihypertensive efficacy does not stem from a negative chronotropic effect; instead, it points toward a reduction in peripheral vascular resistance combined with the preservation of endothelial integrity. This hypothesis is directly reinforced by our functional reactivity data in the mesenteric bed, where NCαA completely prevented both the vasoconstrictor hyper-reactivity to Phe and the impairment of the endothelium-dependent vasodilatory response to ACh. Given that hypertension within the context of MS is heavily aggravated by a marked deficit in nitric oxide (NO) bioavailability [31,32], the comprehensive vascular protection conferred by this specific nanostructured platform strongly implies a significant preservation of the endothelial NO signaling pathway against chronic metabolic degradation.

Extending beyond vascular integrity, MS and chronic hypertension invariably lead to increased cardiac afterload and electrical conduction abnormalities, culminating in ventricular hypertrophy and oxidative stress [31,32]. In our model, this structural remodeling was evidenced by a significant increase in afterload and relative heart weight across all SHR-MS groups. Concomitantly, electrocardiographic recordings in untreated hypertensive controls revealed a significant increase in the depth of the S-wave negative deflection, alongside a significant reduction in EF and an increase in LVESD—well-established electrophysiological and echocardiographic markers of left ventricular overload and hypertrophy [33]. However, a 4-week pharmacological intervention with HCTZ and NCαA (0.3 and 1 mg/kg) effectively mitigated these echocardiographic and electrophysiological alterations, without reversing structural hypertrophy. This discrepancy is consistent with hemodynamic unloading: alleviating afterload and myocardial wall stress restores normal electrical and echocardiographic patterns, whereas the complete regression of established ventricular hypertrophy typically requires extended therapeutic periods [33]. Thus, we hypothesize that a prolonged intervention (>90 days) with HCTZ and NCαA would be necessary to achieve full structural regression. Furthermore, the observed cardioprotective effects of NCαA likely stem from the antioxidant and anti-inflammatory properties inherent to pentacyclic triterpenes; as reported by Del Prado-Audelo et al. [34], Miranda et al. [35], and Wang et al. [36], compounds in this class modulate key pro-inflammatory cytokines, including TNF-α and IL-6, thereby mitigating oxidative stress, reducing myocardial strain, and attenuating cardiovascular remodeling.

Complementing the cardiovascular benefits, serum biochemical analyses and target organ weight evaluations provided essential insights into the systemic efficacy of the treatment, wherein MS induction triggered pronounced oxidative stress and lipid peroxidation—evidenced by depressed serum nitrite and elevated MDA levels—a biochemical phenotype intrinsically linked to impaired NO bioavailability and progressive vascular damage [37,38,39]. Prolonged treatment with NCαA (0.3 and 1 mg/kg) effectively mitigated this pathological state by suppressing lipid peroxidation and restoring circulating nitrite, which, given the ultra-short half-life of NO, serves as a reliable surrogate marker of systemic NO synthesis and bioavailability. However, the concurrent dose-dependent elevation of NT reveals a significant modulation of reactive nitrogen species dynamics; at higher concentrations, excess NO outcompetes endogenous antioxidant defenses and reacts with superoxide anions at near diffusion-controlled rates to generate peroxynitrite. Peroxynitrite and its secondary radical intermediates subsequently mediate the covalent nitration of tyrosine residues, generating NT as a stable footprint of protein oxidation [40]. Thus, while pharmacological intervention with NCαA successfully re-establishes basal NO bioavailability, excessive high-dose generation risks overwhelming endogenous redox buffering systems and shunting excess nitrogen species into the peroxynitrite cascade, culminating in systemic protein nitration.

These protective responses were significantly reflected in the preservation of target organ tissues, as evidenced by relative liver and kidney weights. In the left kidney, the NC and Blank groups exhibited a significant increase in relative weight, indicating renal overload and hypertrophy induced by hypertension and associated metabolic disturbances. However, intervention with HCTZ and all doses of NCαA effectively attenuated this alteration, maintaining renal structural integrity. In parallel, NCαA treatment restored the urinary volume and electrolyte excretion (sodium, potassium, and chloride) that were depressed in the NC group, demonstrating functional improvement without the classic potassium-depleting profile of thiazide diuretics [41].

In the hepatic tissue, the impact of sucrose overload and de novo lipogenesis was evidenced by a marked increase in relative liver weight across the NC, Blank, HCTZ, and low-dose NCαA (0.1 and 0.3 mg/kg) groups [21,22]. Only the treatment with the highest dose of NCαA (1 mg/kg) prevented this increase, keeping the relative liver weight comparable to that of the normotensive (naïve) group. This finding underscores the dose-dependent capacity of the nanoencapsulated formulation to modulate energy metabolism and restrain chronic hepatic lipid accumulation. Taken together, by sustaining hydroelectrolytic balance and protecting both renal and hepatic axes against the structural alterations of MS, these results validate the multifaceted therapeutic reach of this nanostructured platform and warrant future in-depth mechanistic investigations.

Taken together, these findings demonstrate that the nanostructured core–shell formulation NCαA (at doses of 0.3 and 1 mg/kg) represents a multifaceted therapeutic strategy for the integrated management of metabolic syndrome. In addition to preventing excessive weight gain and restoring critical hemodynamic parameters, NCαA conferred significant cardioprotection by preventing electrocardiographic and echocardiographic indicators of left ventricular overload. Furthermore, the formulation increases NO bioavailability and reduces serum levels of MDA, a direct marker of lipid peroxidation. Moreover, NCαA preserved renal functional architecture by maintaining hydroelectrolytic balance without the depleting side-effect profile characteristic of conventional diuretics. By simultaneously counteracting adiposity, systemic hypertension, and endothelial dysfunction, NCαA addresses the clustered pathophysiological mechanisms of MS through a single pharmacological agent, highlighting its translational potential to reduce the clinical burden of polypharmacy and provide a more streamlined, patient-compliant approach to cardiometabolic therapy.

Despite the promising results, the present study has several limitations: (1) Although the data suggest an improvement in redox status, endothelial function, and peripheral vascular resistance, the specific molecular mechanisms by which NCαA exerts its cardioprotective and metabolic effects remain to be elucidated. (2) The absence of in-depth in vivo pharmacokinetic profiles for NCαA prevents a comprehensive understanding of its absorption, distribution, metabolism, and excretion (ADME), necessitating future investigations. (3) HCTZ may not represent an ideal control for key metabolic syndrome features—such as insulin resistance, lipid accumulation, and oxidative stress—unlike standard cardioprotective agents (e.g., statins or renin–angiotensin–aldosterone system inhibitors) against which the therapeutic efficacy of NCαA could be more appropriately benchmarked. (4) While the SHR plus sucrose model is valuable for studying metabolic syndrome, it fails to replicate human atherogenic dyslipidemia, warranting caution when translating these lipid-related findings to clinical contexts. (5) The dose-dependent increase in NT levels induced by higher doses of NCαA indicates a pro-oxidant shift, narrowing the therapeutic window of the nanoformulation and underscoring the necessity of precise dose titration. (6) Finally, the study did not include a direct comparison with other triterpene nanoformulations or alternative bioactive natural compounds, which would have provided a broader perspective on the relative efficacy and advantages of the present formulation.

## 4. Materials and Methods

### 4.1. Drugs and Chemicals

Hydrochlorothiazide, phenylephrine, acetylcholine, sodium nitroprusside, polysorbate 80 (Tween^®^ 80), sucrose, dextrose, ethylenediaminetetraacetic acid (EDTA), α-amyrin (used as a reference standard), and the salts used for physiological solutions (NaCl, KCl, MgSO_4_, NaHCO_3_, KH_2_PO_4_) were purchased from Merck (St. Louis, MO, USA). The α-amyrin used for nanocapsule formulation was kindly provided by the Laboratory of Innovation and Development in Pharmaceutical Technology (LIDETEF) at the Federal University of Amazonas. All chemicals were of analytical grade and used as received.

### 4.2. Synthesis and Characterization of NcαA

Kollicoat MAE 100P^®^ (Basf, São Paulo, SP, Brazil) was used as a film-forming polymer for nanocapsule formation, Tween 80^®^ and Span 80^®^ (Sigma, Chicago, IL, USA) were used as plasticizers. Alpha-amyrin (active) with 98% purity was kindly provided by the Phytochemistry Laboratory, Faculty of Pharmaceutical Sciences, Federal University of Amazonas, Brazil. Isopropyl palmitate (Sigma, Chicago, IL, USA) was used for amyrin solubilization.

#### 4.2.1. Preparation of NCαA

The preparation of NCαA was carried out by the interfacial polymer deposition method followed by solvent displacement, as previously described by Neto et al. [16], a member of our research group, also an author of this work.

#### 4.2.2. NCαA Size, Morphology, and Zeta Potential

The average hydrodynamic diameter (particle size) and polydispersity index (PDI) of the nanocapsules were determined by photon correlation spectroscopy (PCS) using a Zetasizer Nano-ZS instrument (Malvern Instruments, Malvern, UK) [16]. The morphological analysis of the nanocapsules was conducted by scanning electron microscopy (SEM; TESCAN, Brno, Czech Republic). The samples were mounted and sputter-coated with a thin gold layer using a BAL-TEC coater (BAL-TEC AG, Balzers, Liechtenstein). Analysis was performed using a secondary electron detector at an accelerating voltage of 15 kV, a working distance of 8.0 mm, and a magnification of 15.6 kX. The zeta potential was determined by electrophoretic light scattering (ELS) using the same Zetasizer Nano-ZS instrument (Malvern Instruments, Malvern, UK). All analyses were performed in triplicate, and data were expressed as the mean ± standard deviation.

#### 4.2.3. Release Profile

The release profile of αA from nanocapsules was evaluated according to Neto et al. [16]. The assay was performed in a paddle dissolution apparatus (Nova Ética, Vargem Grande Paulista, SP, Brazil) at 37 ± 1 °C using 500 mL of phosphate-buffered saline (PBS, pH 7.4) as the dissolution medium. The percentage of drug released was determined at 0, 10, 25, 50, and 60 min. Quantification was carried out using a calibration curve with α-amyrin (Sigma, Chicago, IL, USA) as an analytical standard, which was previously validated for this purpose (R^2^ = 0.9910, F = 157.11, *p* < 0.0001).

#### 4.2.4. Encapsulation Efficiency

The encapsulation efficiency was determined using a validated high-performance liquid chromatography method [16]. Chromatographic analysis was performed on a Phenomenex Luna C18 column (250 mm × 4.6 mm, 5 µm particle size) using an isocratic mobile phase composed of acetonitrile and water (60:40, *v*/*v*). The flow rate was set at 0.8 mL/min, with a total run time of 15 min. Detection was performed at 200 nm. The concentration of α-amyrin was calculated from a linear calibration curve using analytical-grade α-amyrin as the reference standard.

#### 4.2.5. Shelf-Life Stability Study

Nanocapsule samples stored in amber glass flasks were maintained at 25 ± 2 °C and 65% relative humidity for three months. Particle size, polydispersity index, and zeta-potential were evaluated on days 0, 30, 60, and 90 [16]. All measurements were performed in triplicate, and the data were expressed as the mean ± standard deviation.

### 4.3. In Vivo Pharmacological Assays

#### 4.3.1. Animals

Three-month-old male spontaneously hypertensive rats (SHR) and Wistar-Kyoto (WKY) rats, weighing between 300 and 350 g, were obtained from the Vivarium of the Federal University of Grande Dourados (UFGD). The animals were maintained under controlled conditions at a temperature of 22–25 °C and a 12 h light–dark cycle. Standard chow and chlorinated water were provided ad libitum. For the experimental protocols, *n* = 10 animals per group were used. All experimental procedures followed the Brazilian Guidelines for the Care and Use of Animals for Scientific and Educational Purposes (DBCA) and were approved by the UFGD Animal Use Ethics Committee (Protocol No. 24014, approved on 14 October 2024).

#### 4.3.2. Induction of Metabolic Syndrome (MS)

MS was induced by chronic administration of a 20% sucrose solution in chlorinated water, provided ad libitum for four weeks, following the protocol established by Alvarado-Ibañez et al. [42]. Fasting blood glucose was measured on days 0 and 28 following an 8 h period of food deprivation, using blood collected via tail puncture and a portable glucometer (On Call Plus II).

#### 4.3.3. Experimental Design and Renal Function Evaluation

**Figure 6:**
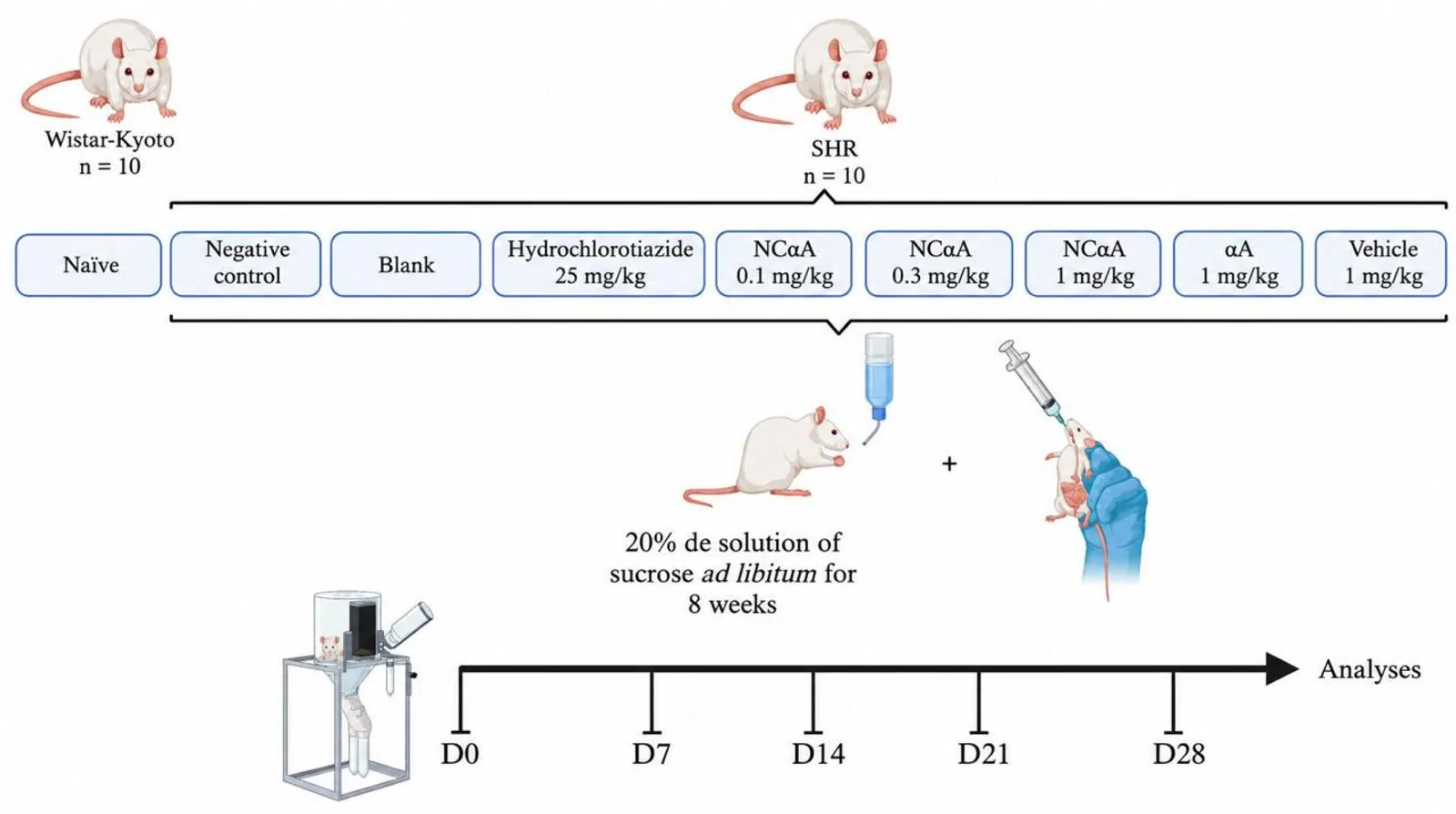
Schematic illustration of the experimental design.

For this protocol (Figure 6), SHR (hypertensive) and WKY (normotensive) rats were divided into the following experimental groups (*n* = 10 per group) and treated daily by gavage for eight weeks as described below:

1. Naïve: WKY rats treated with vehicle (chlorinated water, 0.1 mL/100 g/day);
2. Negative control: SHRs with sucrose-induced MS, treated with vehicle (filtered water, 0.1 mL/100 g/day);
3. Blank: SHRs with sucrose-induced MS, treated with blank polymeric nanocapsule group (filtered water, 0.1 mL/100 g/day);
4. Positive control: SHRs with sucrose-induced MS, treated with hydrochlorothiazide (25 mg/kg/day);
5. NCαA: SHRs with sucrose-induced MS, treated with α-amyrin-loaded nanocapsules at three different doses (0.1, 0.3, or 1 mg/kg/day);
6. αA: SHRs with sucrose-induced MS, treated with α-amyrin (1 mg/kg/day).

Weekly, to promote body salt and water uniformity, all groups received a 0.9% sodium chloride (NaCl) solution (5 mL/100 g) one hour before the start of diuresis. On days 0 and 28, 24 h urine samples were collected using metabolic cages equipped with graduated cylinders. Urinary pH, conductivity, density, and concentrations of sodium, potassium, chloride, and bicarbonate were determined. To monitor metabolic parameters, initial and final capillary glycemia was measured via tail vein puncture on days 1 and 28. In addition, mortality rates were monitored daily, and body weight was recorded weekly using a digital scale. (Ohaus, Parsippany, NJ, USA). At the end of the experimental period (day 29), all animals were anesthetized with isoflurane (2–3%) via inhalation. Subsequently, all groups were subjected to the experimental procedures described below.

#### 4.3.4. Electrocardiogram

After the induction of anesthesia, the animals were placed in a supine position. Four electrodes (right limb, RL; left limb, LL; right arm, RA; and left arm, LA) were attached to the wrists and ankles, using a small amount of conductive gel to enhance electrical conductance. Following a 5 min acclimatization period, electrocardiographic signals were recorded for 5 min using an electrocardiograph (WinCardio, Micromed, Brasília, Brazil).

#### 4.3.5. Transthoracic Echocardiography

Transthoracic echocardiographic assessments were conducted to evaluate cardiac structure and function using an Eco1 Hipermedic system equipped with a high-frequency transducer (Hipermedic, Porto Alegre, RS, Brazil). Following ECG measurements, rats were placed on a temperature-controlled heating pad (37 ± 0.5 °C), and the anterior precordial region was shaved and covered with acoustic coupling gel. Two-dimensional guided M-mode tracings were acquired from the parasternal short-axis view at the level of the papillary muscles. Pulsed-wave Doppler was aligned at the mitral valve leaflets in the apical four-chamber view to record early (E) and late (A) transmitral filling velocities, deceleration time (DT), and the E/A ratio to assess diastolic function. The following parameters were evaluated: left ventricular end-diastolic diameter (LVEDD), left ventricular end-systolic diameter (LVESD), end-diastolic volume (EDV), end-systolic volume (ESV), ejection fraction (EF), and stroke volume (SV). All recordings were averaged across three to five consecutive cardiac cycles by a single blinded operator to ensure measurement consistency across experimental groups.

#### 4.3.6. Measurement of Blood Pressure and Heart Rate

Following electrocardiography, a subcutaneous bolus injection of heparin (20 UI) was administered to all animals. Subsequently, the left carotid artery was isolated and cannulated with a polyethylene catheter connected to a pressure transducer coupled to a computerized data acquisition system (LabChart, v 8.0, ADInstruments, Sydney, Australia). Systolic blood pressure, DBP, MAP, and HR were recorded for 5 min.

#### 4.3.7. Evaluation of Mesenteric Vascular Bed Reactivity

Following blood pressure measurement and while the animals were under deep anesthesia, the MVB was cannulated and gently flushed with physiological saline solution (PSS; 119 mM NaCl, 4.7 mM KCl, 2.4 mM MgSO_4_, 25.9 mM NaHCO_3_, 1.2 mM KH_2_PO_4_, 11.1 mM dextrose, and 0.3 mM EDTA; pH 7.4) according to a previously described technique [43,44]. The MVB was then isolated and maintained at 37 °C, aerated with carbogen (95% O_2_/5% CO_2_), and perfused with PSS using a peristaltic pump (Tecnopon; Piracicaba, SP, Brazil) at a constant flow rate of 4 mL/min. Changes in perfusion pressure were recorded using a pressure transducer connected to a signal amplifier and coupled to a data acquisition system (PowerLab, LabChart v 8.0, ADInstruments, Sydney, Australia). Following a 30 min stabilization period, the functional integrity of the preparation was verified by a bolus injection of KCl (120 nmol). Subsequently, dose–response administrations of phenylephrine (1, 3, 10, and 30 nmol) were generated. After another stabilization period, the preparations were pre-contracted by continuous infusion of PSS containing 30 μM phenylephrine to elevate perfusion pressure to approximately 90–100 mm Hg. Once a stable plateau was achieved, dose–response administrations of acetylcholine (1, 3, 10, and 30 nmol) and sodium nitroprusside (3, 10, 30, and 100 nmol) were performed. The vascular reactivity responses were compared among the experimental groups.

#### 4.3.8. Blood Collection and Quantification of Serum Biochemical Markers

On day 29, following blood pressure measurements and prior to the removal of the mesenteric vascular beds, blood samples (4–5 mL) were obtained from the previously isolated left carotid artery. Serum was separated by centrifugation at 1500× *g* for 15 min. Serum levels of alanine aminotransferase (ALT), aspartate aminotransferase (AST), urea, creatinine, cardiac troponin T (cTNT), sodium, and potassium were measured using an automated biochemical analyzer (Roche Cobas Integra 400 Plus). Serum insulin and nitrotyrosine (NT) levels were determined using commercial enzyme immunoassay kits (MyBioSource, San Diego, CA, USA). Malondialdehyde (MDA) levels were measured using a commercial assay kit (Cayman Chemical, Ann Arbor, MI, USA). Plasma nitrite levels were determined by the enzymatic reduction of nitrate, according to a previously described technique [45]. Angiotensin-converting enzyme (ACE) activity and serum aldosterone levels were also quantified. Finally, the animals were euthanized by isoflurane overdose in a saturation chamber (concentration above 40%), following the Guidelines for Euthanasia Practice of the National Council for the Control of Animal Experimentation (CONCEA) and Resolution No. 1000/2013 of the Federal Council of Veterinary Medicine (CFMV).

#### 4.3.9. Relative Organ Weights

At the end of the experimental period and immediately following euthanasia, the animals were weighed to obtain their final body weight. Subsequently, the organs of interest (heart, kidneys, liver, and spleen) were carefully removed, dissected to clear any adjacent adipose or connective tissue, and rinsed with physiological saline solution. Excess moisture was removed using absorbent paper (Scott; São Paulo, SP, Brazil), and the absolute weight of each organ was determined using a precision analytical balance (Mettler Toledo; Greifensee, Zurich, Switzerland). The relative weight of each organ (somatic index) was calculated using the following formula: Relative organ weight (%) = (absolute organ weight [g])/final body weight [g]) × 100. The results were expressed as a percentage of body weight or as grams of tissue per 100 g of body weight (g/100 g).

#### 4.3.10. Histopathological Analyses

Samples of the liver, left kidney, heart (left ventricle), and aorta (aortic arch) were collected, cleaned, fixed in 10% buffered formalin, dehydrated, and embedded in paraffin. Five-micrometer (5 µm) sections were stained with hematoxylin and eosin (H&E). Histopathological evaluation focused on cellular damage, fibrosis, and inflammation, using the following validated semi-quantitative scoring system: score 0, absent lesion/normal tissue; score 1, isolated focus of lesion/inflammation (<10% of the area); score 2, moderate multifocal lesions (10–30% of the area); score 3, extensive confluent lesions (30–50% of the area); and score 4, diffuse and severe damage (>50% of the area). Reproducibility and reliability were ensured through an intra-observer protocol in which the same pathologist evaluated the slides at two distinct time points (separated by a 2-week interval) while blinded to sample identity. A combined qualitative and quantitative analysis of the organs was performed using a Nikon ECLIPSE Si microscope (Nikon Corporation; Tokyo, Japan) equipped with Motic Images Plus 2.0 software.

### 4.4. Statistical Analysis

Data were analyzed for homogeneity of variance and normality. Statistical analyses were performed using one-way analysis of variance (ANOVA) followed by Tukey’s post hoc test. The results were expressed as mean ± standard deviation (SD), and *p* values less than 0.05 were considered statistically significant. All analyses were performed and graphs were generated using GraphPad Prism software (v10.0, GraphPad Software, LLC, San Diego, CA, USA).

## 5. Conclusions

This study demonstrates that polymeric nanoencapsulation successfully overcomes the critical biopharmaceutical bottlenecks of α-amyrin, providing an innovative nanomedicine platform for managing metabolic syndrome and its associated cardiovascular complications. Physicochemical characterization confirmed the successful engineering of core–shell nanocapsules (NCαA) exhibiting a submicron hydrodynamic diameter, narrow unimodal size distribution, spherical morphology, and a strongly negative zeta potential, which collectively ensured robust colloidal stability and prevented particle agglomeration. Furthermore, the high encapsulation efficiency coupled with sustained in vitro dissolution kinetics confirmed that the nanocarrier maximized drug payload delivery and release performance.

In vivo, prolonged oral treatment with NCαA—particularly at intermediate and higher doses—prevented excessive body weight gain, normalized arterial pressures (systolic, diastolic, and mean), and attenuated both electrocardiographic (S-wave deflection) and echocardiographic (ejection fraction and left ventricular end-systolic diameter) markers of cardiac overload. Furthermore, NCαA conferred comprehensive vascular protection by restoring nitric oxide bioavailability, preventing endothelial dysfunction, reducing malondialdehyde levels, and preserving renal hydroelectrolytic balance without inducing electrolyte wasting.

Nevertheless, the significant dose-dependent elevation of serum nitrotyrosine observed at higher doses indicates a pro-oxidative shift driven by peroxynitrite formation. This phenomenon highlights a potentially narrow therapeutic window and emphasizes the absolute necessity for precise dosage titration to avoid overwhelming endogenous redox buffering systems while maintaining therapeutic efficacy.

Finally, although these findings highlight NCαA as a multifaceted and promising candidate for cardiometabolic disorders, several limitations must be acknowledged to avoid over-interpreting the experimental data. The specific downstream molecular pathways mediating its cardioprotective actions remain to be fully characterized, and comprehensive in vivo pharmacokinetic profiles are still lacking. Furthermore, the spontaneously hypertensive rat model with sucrose overload does not fully recapitulate human atherogenic dyslipidemia, hydrochlorothiazide serves as a hemodynamic rather than an ideal metabolic benchmark, and direct comparisons with other bioactive triterpenoid nanoformulations were not performed. Addressing these constraints, along with implementing advanced analytical techniques to distinguish core-entrapped from surface-adsorbed fractions, will be essential in future studies to fully establish the clinical translational value of NCαA.

## Figures and Tables

**Table 1:** Shelf-life stability of α-amyrin-loaded nanocapsules (NcαA).

Time (Day)	Size (nm)	Polydispersity Index	Zeta-Potential (mV)
0	195.10 ± 1.55	0.144 ± 0.005	−52.60 ± 1.25
30	194.22 ± 1.87	0.147 ± 0.022	−55.91 ± 1.57
60	194.26 ± 1.48	0.146 ± 0.010	−53.50 ± 2.01
90	195.36 ± 1.37	0.144 ± 0.012	−56.65 ± 1.33

Statistical analyses were performed using one-way ANOVA followed by Tukey’s test. All analyses were performed in triplicate. Data are presented as mean ± standard deviation.

**Table 2:** Effect of α-amyrin-loaded polymeric nanocapsules (NCαA) on urine volume and renal excretion of sodium (Na^+^), potassium (K^+^), chloride (Cl^−^), and bicarbonate (HCO_3_^−^) in different experimental groups on days 1 and 28.

Parameter	Naïve	NC	HCTZ 25 mg/kg	NCαA 0.1 mg/kg	NCαA 0.3 mg/kg	NCαA 1 mg/kg	αA 1 mg/kg
Day 1							
24 h urine sample/100 g	6.14 ± 1.8	7.21 ± 1.4	11.12 ± 2.2 ^a^	6.21 ± 1.8	6.84 ± 1.4	6.03 ± 1.9	7.18 ± 1.3
Na^+^ (mmol/L)	103.1 ± 7.7	112.2 ± 8.1	150.2 ± 8.7 ^a^	114.2 ± 7.1	116.5 ± 8.2	111.1 ± 7.5	105.4 ± 7.6
K^+^ (mmol/L)	32.1 ± 3.21	33.6 ± 4.01	59.1 ± 3.6 ^a^	27.3 ± 4.1	30.1 ± 3.4	32.9 ± 3.5	31.5 ± 3.7
Cl^−^ (mmol/L)	142.1 ± 11.6	140.2 ± 11.8	177.2 ± 13.5 ^a^	140.7 ± 11.6	135.1 ± 13.5	137.3 ± 12.1	137.7 ± 11.0
HCO_3_^−^ (mmol/L)	210.1 ± 22.7	213.1 ± 19.2	221.3 ± 19.1	217.3 ± 21.6	222.7 ± 19.3	216.5 ± 19.7	200.3 ± 18.0
Day 28							
24 h urine sample/100 g	7.46 ± 2.2	3.11 ± 1.4 ^a^	7.98 ± 1.6 ^b^	3.22 ± 1.7 ^a^	8.83 ± 2.8 ^b^	7.24 ± 0.8 ^b^	2.92 ± 1.32 ^a^
Na^+^ (mmol/L)	109.3 ± 8.2	65.7 ± 5.1 ^a^	100.7 ± 7.1 ^b^	78.2 ± 6.7 ^a^	114.5 ± 9.2 ^b^	104.5 ± 8.1 ^b^	61.7 ± 4.7 ^a^
K^+^ (mmol/L)	29.3 ± 4.01	15.1 ± 3.5 ^a^	27.3 ± 4.1 ^b^	16.4 ± 4.4 ^a^	30.6 ± 3.9 ^b^	27.6 ± 4.2 ^b^	14.1 ± 3.2 ^a^
Cl^−^ (mmol/L)	135.3 ± 12.7	100.1 ± 6.2 ^a^	140.4 ± 13.5 ^b^	107.6 ± 5.9 ^a^	147.1 ± 12.7 ^b^	146.8 ± 11.9 ^b^	94.0 ± 5.8 ^a^
HCO_3_^−^ (mmol/L)	221.3 ± 25.1	233.4 ± 21.6	227.7 ± 22.5	228.6 ± 19.4	236.5 ± 23.1	217.5 ± 19.8	229.4 ± 20.3

Statistical analyses were performed using one-way ANOVA followed by Tukey’s test. Data are presented as mean ± standard deviation. ^a^ *p* ≤ 0.05 indicates statistical significance compared to the naïve group, and ^b^ *p* ≤ 0.05 compared to the negative control group (NC). HCTZ: hydrochlorothiazide; αA: α-amyrin.

**Table 3:** Effects of α-amyrin-loaded polymeric nanocapsules (NCαA) on body weight gain and electrocardiographic parameters in different experimental groups on day 29.

Parameter	Naïve	NC	HCTZ 25 mg/kg	NCαA 0.1 mg/kg	NCαA 0.3 mg/kg	NCαA 1 mg/kg	αA 1 mg/kg
Weight gain (g)	31.14 ± 3.1	47.00 ± 4.9 ^a^	33.11 ± 2.9 ^b^	45.06 ± 4.9 ^a^	31.11 ± 4.2 ^b^	24.14 ± 3.1 ^b^	42.30 ± 4.4 ^a^
Interval PR (ms)	36.11 ± 4.1	38.14 ± 2.9	35.50 ± 2.3	38.86 ± 2.6	38.67 ± 2.7	38.33 ± 2.2	34.32 ± 2.61
Interval QRS (ms)	41.11 ± 2.7	42.00 ± 2.9	39.00 ± 1.9	38.57 ± 1.9	43.17 ± 5.1	38.33 ± 1.8	37.80 ± 2.6
Interval QT (ms)	75.63 ± 4.8	77.86 ± 4.5	77.50 ± 3.2	71.57 ± 3.8	82.17 ± 7.8	77.50 ± 4.8	70.07 ± 4.0
Interval QTc (ms)	179.3 ± 5.9	178.0 ± 5.6	187.5 ± 11.8	164.1 ± 7.9	191.7 ± 21.0	173.7 ± 16.1	170.2 ± 5.0
Wave P (mV)	0.05 ± 0.006	0.06 ± 0.005	0.05 ± 0.007	0.04 ± 0.007	0.04 ± 0.005	0.06 ± 0.007	0.05 ± 0.004
Wave Q (mV)	−0.01 ± 0.003	−0.01 ± 0.004	−0.02 ± 0.004	−0.01 ± 0.002	−0.02 ± 0.005	−0.02 ± 0.008	−0.009 ± 0.004
Wave R (mV)	0.25 ± 0.02	0.28 ± 0.02	0.24 ± 0.02	0.26 ± 0.02	0.29 ± 0.03	0.22 ± 0.02	0.25 ± 0.02
Wave S (mV)	−0.02 ± 0.01	−0.06 ± 0.01 ^a^	−0.02 ± 0.01 ^b^	−0.05 ± 0.01 ^a^	−0.02 ± 0.01 ^b^	−0.02 ± 0.01 ^b^	−0.05 ± 0.01 ^a^

Statistical analyses were performed using one-way ANOVA followed by Tukey’s test. Data are presented as mean ± standard deviation. ^a^ *p* ≤ 0.05 indicates statistical significance compared to the naïve group, and ^b^ *p* ≤ 0.05 compared to the negative control group (NC). HCTZ: hydrochlorothiazide; αA: α-amyrin.

**Table 4:** Effects of α-amyrin-loaded polymeric nanocapsules (NCαA) on echocardiographic parameters in different experimental groups on day 29.

Parameter	Naïve	NC	HCTZ 25 mg/kg	NCαA 0.1 mg/kg	NCαA 0.3 mg/kg	NCαA 1 mg/kg	αA 1 mg/kg
EDV (mL)	0.39 ± 0.05	0.35 ± 0.07	0.38 ± 0.08	0.37 ± 0.09	0.33 ± 0.06	0.39 ± 0.07	0.38 ± 0.06
ESV (mL)	0.09 ± 0.02	0.12 ± 0.02	0.11 ± 0.03	0.10 ± 0.02	0.11 ± 0.03	0.12 ± 0.03	0.10 ± 0.02
EF (%)	85.8 ± 3.3	70.3 ± 3.4 ^a^	86.9 ± 3.1 ^b^	69.2 ± 3.8 ^a^	86.9 ± 3.9 ^b^	87.1 ± 4.3 ^b^	71.4 ± 4.2 ^a^
LVEDD (mm)	3.63 ± 0.31	3.86 ± 0.22	3.50 ± 0.25	3.57 ± 0.28	3.67 ± 0.34	3.50 ± 0.38	3.67 ± 0.31
LVESD (mm)	6.25 ± 0.12	7.28 ± 0.13 ^a^	6.24 ± 0.14 ^b^	7.26 ± 0.13 ^a^	6.29 ± 0.13 ^b^	6.22 ± 0.04 ^b^	7.25 ± 0.12 ^a^
SV (mL)	0.27 ± 0.07	0.29 ± 0.08	0.26 ± 0.07	0.25 ± 0.06	0.28 ± 0.07	0.27 ± 0.07	0.25 ± 0.08

Statistical analyses were performed using one-way ANOVA followed by Tukey’s test. Data are presented as mean ± standard deviation. ^a^ *p* ≤ 0.05 indicates statistical significance compared to the naïve group, and ^b^ *p* ≤ 0.05 compared to the negative control group (NC). EDV: end-diastolic volume; ESV: end-systolic volume; EF: ejection fraction; HCTZ: hydrochlorothiazide; LVEDD: left ventricular end-diastolic diameter; LVESD: left ventricular end-systolic diameter; SV: stroke volume; αA: α-amyrin.

**Table 5:** Effects of α-amyrin-loaded polymeric nanocapsules (NCαA) on systolic blood pressure (SBP), diastolic blood pressure (DBP), mean arterial pressure (MAP), and heart rate in different experimental groups on day 29.

Parameter	Naïve	NC	HCTZ 25 mg/kg	NCαA 0.1 mg/kg	NCαA 0.3 mg/kg	NCαA 1 mg/kg	αA 1 mg/kg
PAS (mm Hg)	128.2 ± 8.2	153.1 ± 8.3 ^a^	124.8 ± 4.3 ^b^	154.12 ± 5.1 ^a^	127.11 ± 6.8 ^b^	125.22 ± 6.2 ^b^	142.38 ± 7.72 ^a^
PAD (mm Hg)	78.91 ± 7.4	94.51 ± 8.2 ^a^	77.22 ± 7.1 ^b^	93.33 ± 6.3 ^a^	74.21 ± 5.4 ^b^	73.77 ± 5.5 ^b^	87.89 ± 7.63 ^a^
PAM (mm Hg)	92.7 ± 6.4	107.12 ± 5.6 ^a^	91.0 ± 7.6 ^b^	108.03 ± 14.3 ^a^	93.33 ± 9.1 ^b^	92.22 ± 9.2 ^b^	99.62 ± 5.21 ^a^
FC (bpm)	352.60 ± 74.8	388.8 ± 17.6	338.8 ± 20.3	317.2 ± 31.3	292.8 ± 18.9	272.8 ± 10.4	361.58 ± 16.37

Statistical analyses were performed using one-way ANOVA followed by Tukey’s test. Data are presented as mean ± standard deviation. ^a^ *p* ≤ 0.05 indicates statistical significance compared to the naïve group, and ^b^ *p* ≤ 0.05 compared to the negative control group (NC). Bpm: beats per minute; HCTZ: hydrochlorothiazide; αA: α-amyrin.

**Table 6:** Effects of α-amyrin-loaded polymeric nanocapsules (NCαA) on mesenteric vascular reactivity in different experimental groups on day 29.

Drug	Naïve	NC	HCTZ 25 mg/kg	NCαA 0.1 mg/kg	NCαA 0.3 mg/kg	NCαA 1 mg/kg	αA 1 mg/kg
Phe (nmol)							
1	3.29 ± 2.6	1.03 ± 2.3	1.39 ± 1.6	0.94 ± 1.3	2.58 ± 3.2	1.85 ± 1.9	0.96 ± 2.1
3	26.4 ± 15.5	33.4 ± 12.1	31.2 ± 11.4	34.3 ± 13.9	31.4 ± 13.5	30.3 ± 11.2	31.1 ± 11.3
10	55.0 ± 13.8	109.2 ± 13.2 ^a^	54.7 ± 17.5 ^b^	104.9 ± 8.5 ^a^	52.0 ± 3.9 ^b^	53.4 ± 8.1 ^b^	101.6 ± 12.3 ^a^
30	105.1 ± 21.7	156.6 ± 33.9 ^a^	105.7 ± 26.6 ^b^	153.6 ± 23.9 ^a^	101.2 ± 35.3 ^b^	106.4 ± 31.5 ^b^	145.6 ± 31.5 ^a^
ACh (nmol)							
1	−29.81 ± 5.5	−28.9 ± 5.9	−31.4 ± 6.2	−27.3 ± 4.9	−27.6 ± 6.7	−25.1 ± 4.1	−26.9 ± 5.5
3	−37.07 ± 7.2	−39.3 ± 6.7	−33.6 ± 7.9	−32.0 ± 6.7	−38.9 ± 6.1	−32.6 ± 6.2	−36.5 ± 6.2
10	−52.12 ± 6.7	−50.1 ± 7.6	−54.7 ± 7.3	−55.9 ± 7.8	−50.1 ± 5.9	−51.4 ± 6.5	−46.6 ± 7.1
30	−84.38 ± 8.8	−53.7 ± 6.7 ^a^	−88.2 ± 8.4 ^b^	−44.6 ± 6.3 ^a^	−86.8 ± 7.3 ^b^	−87.7 ± 8.5 ^b^	−49.9 ± 6.2 ^a^
SNP (nmol)							
3	−24.2 ± 4.1	−26.4 ± 8.0	−25.1 ± 6.1	−23.0 ± 5.6	−25.4 ± 7.7	−23.6 ± 4.3	−24.6 ± 7.4
10	−35.2 ± 7.2	−33.4 ± 6.6	−38.9 ± 6.5	−35.7 ± 6.2	−39.6 ± 5.8	−37.5 ± 5.9	−31.1 ± 6.1
30	−55.5 ± 9.9	−58.5 ± 6.2	−50.7 ± 7.5	−54.4 ± 6.6	−54.1 ± 8.9	−53.7 ± 7.5	−54.4 ± 5.8
100	−77.4 ± 8.0	−78.9 ± 9.9	−79.5 ± 8.7	−78.1 ± 7.4	−79.7 ± 8.5	−75.7± 7.4	−73.4 ± 9.2

Statistical analyses were performed using one-way ANOVA followed by Tukey’s test. Data are presented as mean ± standard deviation. ^a^ *p* ≤ 0.05 indicates statistical significance compared to the naïve group, and ^b^ *p* ≤ 0.05 compared to the negative control group (NC). Ach: acetylcholine; HCTZ: hydrochlorothiazide; SNP: sodium nitroprusside; Phe: phenylephrine; αA: α-amyrin.

**Table 7:** Effects of α-amyrin-loaded polymeric nanocapsules (NCαA) on serum biochemical parameters in different experimental groups on day 29.

Parameter	Naïve	NC	HCTZ 25 mg/kg	NCαA 0.1 mg/kg	NCαA 0.3 mg/kg	NCαA 1 mg/kg	αA 1 mg/kg
AST (U/L)	39.4 ± 5.3	35.2 ± 4.8	36.3 ± 5.2	32.1 ± 6.0	30.5 ± 5.2	33.9 ± 5.9	32.7 ± 4.5
ALT (U/L)	32.2 ± 4.1	32.2 ± 4.1	32.2 ± 4.1	32.2 ± 4.1	32.2 ± 4.1	32.2 ± 4.1	29.9 ± 3.8
Triglycerides	72.4 ± 8.4	141.2 ± 9.1 ^a^	143.5 ± 8.4 ^a^	145.1 ± 9.2 ^a^	146.8 ± 7.7 ^a^	148.4 ± 8.8 ^a^	149.7 ± 9.0 ^a^
Total cholesterol	68.4 ± 6.8	72.1 ± 7.2	75.6 ± 7.6	78.3 ± 7.8	81.5 ± 8.2	84.2 ± 8.4	80.6 ± 7.1
LDL-cholesterol	20.2 ± 2.4	19.5 ± 2.7	21.1 ± 2.8	20.4 ± 2.0	22.3 ± 2.2	20.6 ± 2.5	21.4 ± 2.1
HDL-cholesterol	45.2 ± 4.1	46.5 ± 4.3	47.1 ± 4.5	46.8 ± 4.7	48.4 ± 4.8	50.2 ± 5.0	49.6 ± 6.6
cTNT (µg/L)	0.11 ± 0.02	0.12 ± 0.03	0.12 ± 0.02	0.11 ± 0.03	0.13 ± 0.03	0.12 ± 0.02	0.12 ± 0.04
Nitrite (µM)	4.84 ± 0.8	2.62 ± 0.8 ^a^	2.77 ± 0.7 ^a^	3.51 ± 0.8	4.92 ± 0.9 ^b^	5.34 ± 1.0 ^b^	2.74 ± 0.7 ^a^
NT (µmol/L)	0.010 ± 0.004	0.013 ± 0.003	0.014 ± 0.004	0.015 ± 0.004	0.026 ± 0.003 ^ab^	0.029 ± 0.004 ^ab^	0.012 ± 0.003
MDA (µmol/L)	1.43 ± 0.3	2.52 ± 0.4 ^a^	2.40 ± 0.5 ^a^	1.92 ± 0.5	1.37 ± 0.1 ^b^	1.28 ± 0.1 ^b^	2.44 ± 0.4 ^a^
Na^+^ (mg/dL)	125.1 ± 8.3	129.3 ± 6.4	127.3 ± 7.1	123.8 ± 7.2	124.7 ± 6.4	123.3 ± 8.5	120.2 ± 6.0
K^+^ (mg/dL)	5.5 ± 1.4	5.4 ± 1.2	5.3 ± 1.3	5.0 ± 1.0	5.2 ± 1.3	5.9 ± 1.1	5.0 ± 1.1
Urea (mg/dL)	27.1 ± 3.7	30.3 ± 2.2	28.4 ± 3.1	29.7 ± 3.9	26.6 ± 3.4	25.7 ± 3.2	28.2 ± 2.0
Creatinine (mg/dL)	0.75 ± 0.1	1.02 ± 0.2	0.85 ± 0.2	0.92 ± 0.2	0.90 ± 0.2	0.81 ± 0.2	0.98 ± 0.2
Insulin (ng/mL)	0.15 ± 0.08	1.76 ± 0.40 ^a^	1.82 ± 0.65 ^a^	1.88 ± 0.59 ^a^	1.79 ± 0.46 ^a^	1.84 ± 0.51 ^a^	1.77 ± 0.49 ^a^
Aldosterone (pg/mL)	122.4 ± 7.6	120.3 ± 9.2	117.6 ± 8.2	135.5 ± 10.9	120.4 ± 9.8	117.2 ± 8.4	117.9 ± 8.6
ACE activity (nmol/min/mL)	90.3 ± 7.2	95.3 ± 8.6	97.3 ± 8.9	99.2 ± 9.9	95.1 ± 9.1	92.2 ± 8.8	88.6 ± 8.0

Statistical analyses were performed using one-way ANOVA followed by Tukey’s test. Data are presented as mean ± standard deviation. ^a^ *p* ≤ 0.05 indicates statistical significance compared to the naïve group, and ^b^ *p* ≤ 0.05 compared to the negative control group (NC). ALT: alanine aminotransferase; AST: aspartate aminotransferase; cTNT: cardiac troponin T; ACE: angiotensin-converting enzyme; HCTZ: hydrochlorothiazide; HDL-cholesterol: high-density lipoprotein cholesterol; LDL-cholesterol: low-density lipoprotein cholesterol; MDA: malondialdehyde; NT: nitrotyrosine; αA: α-amyrin.

**Table 8:** Effects of α-amyrin-loaded polymeric nanocapsules (NCαA) on relative target organ weight in different experimental groups on day 29.

Parameter	Naïve	NC	HCTZ 25 mg/kg	NCαA 0.1 mg/kg	NCαA 0.3 mg/kg	NCαA 1 mg/kg	αA 1 mg/kg
Heart	0.24 ± 0.006	0.36 ± 0.02 ^a^	0.35 ± 0.009 ^a^	0.36 ± 0.01 ^a^	0.36 ± 0.006 ^a^	0.35 ± 0.008 ^a^	0.33 ± 0.02 ^a^
Spleen	0.15 ± 0.005	0.16 ± 0.004	0.15 ± 0.004	0.16 ± 0.005	0.16 ± 0.003	0.16 ± 0.004	0.15 ± 0.004
Right kidney	0.32 ± 0.02	0.33 ± 0.01	0.32 ± 0.009	0.32 ± 0.007	0.31 ± 0.01	0.30 ± 0.007	0.31 ± 0.01
Left kidney	0.28 ± 0.01	0.32 ± 0.009 ^a^	0.30 ± 0.008	0.31 ± 0.008	0.30 ± 0.008	0.31 ± 0.01	0.32 ± 0.01 ^a^
Liver	2.65 ± 0.09	3.32 ± 0.14 ^a^	3.32 ± 0.14 ^a^	3.47 ± 0.11 ^a^	3.27 ± 0.16 ^a^	3.16 ± 0.09	3.19 ± 0.13 ^a^

Statistical analyses were performed using one-way ANOVA followed by Tukey’s test. Data are presented as mean ± standard deviation. ^a^ *p* ≤ 0.05 indicates statistical significance compared to the naïve group. HCTZ: hydrochlorothiazide; αA: α-amyrin.

## Data Availability

The original contributions presented in this study are included in the article/Supplementary Materials. Further inquiries can be directed to the corresponding author.

## Supplementary Materials

The following supporting information can be downloaded at: https://www.mdpi.com/article/10.3390/ph19101599/s1, Table S1. Comprehensive functional, hemodynamic, electrocardiographic, vascular, biochemical, and biometric parameters of SHR rats with sucrose-induced metabolic syndrome receiving blank polymeric nanocapsules (Blank group); Figure S1. Representative histological images of the liver, left kidney, left ventricle, and aortic arch from the naive, negative control, blank, HCTZ, α-amyrin-loaded nanocapsules, and αA (1 mg/kg; free α-amyrin) groups. H&E: hematoxylin and eosin stain.
