# Supplementary material for: Cardioprotective Effect of Nanoencapsulated α-Amyrin in Spontaneously Hypertensive Rats with Metabolic Syndrome
Source: Pharmaceuticals (Basel). 2026 Oct 9;19(10):1599. doi: 10.3390/ph19101599 (PMC13708067; doi:10.3390/ph19101599)
Supplement: Supplementary file 1 [file pharmaceuticals-19-01599-s001.zip › pharmaceuticals-4571540-supplementary.pdf]

Supplementary Material

Table S1. Comprehensive functional, hemodynamic, electrocardiographic, vascular, biochemical, and biometric parameters of SHR rats with sucrose-induced metabolic syndrome receiving blank polymeric nanocapsules (Blank group).

Parameter	Blank Nanocapsules (Blank)
Renal Function & Hydroelectrolytic Balance (Day 1)	
24-h urine volume (mL/100 g)	7.15 ± 1.3
Urinary Na^+ (mmol/L)	110.8 ± 7.9
Urinary K^+ (mmol/L)	34.2 ± 3.8
Urinary Cl^- (mmol/L)	138.9 ± 11.2
Urinary HCO_3^- (mmol/L)	211.5 ± 18.7
Renal Function & Hydroelectrolytic Balance (Day 28)	
24-h urine volume (mL/100 g)	**3.08 ± 1.2**[a]
Urinary Na^+ (mmol/L)	**64.2 ± 4.9**[a]
Urinary K^+ (mmol/L)	**14.8 ± 3.3**[a]
Urinary Cl^- (mmol/L)	**98.4 ± 6.0**[a]
Urinary HCO_3^- (mmol/L)	**230.1 ± 20.4**[a]
Biometric & Electrocardiographic Parameters (Day 29)	
Body weight gain (g)	**46.25 ± 4.6**[a]
PR interval (ms)	37.82 ± 2.7
QRS interval (ms)	41.50 ± 2.8
QT interval (ms)	76.90 ± 4.2
QTc interval (ms)	176.4 ± 5.8
P wave (mV)	0.058 ± 0.006
Q wave (mV)	-0.011 ± 0.004
R wave (mV)	0.27 ± 0.02
S wave (mV)	**-0.058 ± 0.01**[a]
Transthoracic Echocardiography (Day 29)	
End-diastolic volume, EDV (mL)	0.36 ± 0.06
End-systolic volume, ESV (mL)	0.11 ± 0.02
Ejection fraction, EF (%)	**70.8 ± 3.6**[a]
Left ventricular end-diastolic diameter, LVEDD (mm)	3.81 ± 0.24
Left ventricular end-systolic diameter, LVESD (mm)	**7.24 ± 0.12**[a]
Stroke volume, SV (mL)	0.28 ± 0.07
Hemodynamic Parameters (Day 29)	
Systolic blood pressure, SBP (mm Hg)	**152.4 ± 7.9**[a]
Diastolic blood pressure, DBP (mm Hg)	**93.8 ± 7.8**[a]
Mean arterial pressure, MAP (mm Hg)	**106.4 ± 6.1**[a]
Heart rate, HR (bpm)	382.5 ± 18.4
Mesenteric Vascular Bed Reactivity (Day 29)	
Phenylephrine 1 nmol (mm Hg)	1.12 ± 2.1
Phenylephrine 3 nmol (mm Hg)	32.8 ± 11.5
Phenylephrine 10 nmol (mm Hg)	**107.5 ± 12.8**[a]
Phenylephrine 30 nmol (mm Hg)	**154.2 ± 31.0**[a]
Acetylcholine 1 nmol (mm Hg)	-28.1 ± 5.4
Acetylcholine 3 nmol (mm Hg)	-38.4 ± 6.2
Acetylcholine 10 nmol (mm Hg)	-49.5 ± 7.0
Acetylcholine 30 nmol (mm Hg)	**-52.4 ± 6.5**[a]
Sodium nitroprusside 3 nmol (mm Hg)	-25.8 ± 7.5
Sodium nitroprusside 10 nmol (mm Hg)	-34.1 ± 6.2
Sodium nitroprusside 30 nmol (mm Hg)	-57.2 ± 6.0

Sodium nitroprusside 100 nmol (mm Hg)	-77.5 ± 9.1
Serum Biochemical Parameters (Day 29)	
AST (U/L)	34.6 ± 4.5
ALT (U/L)	31.8 ± 3.9
Triglycerides (mg/dL)	**144.5 ± 8.9**[a]
Total cholesterol (mg/dL)	73.8 ± 7.0
LDL-cholesterol (mg/dL)	20.1 ± 2.4
HDL-cholesterol (mg/dL)	47.2 ± 4.4
cTNT (µg/L)	0.12 ± 0.02
Nitrite (µM)	**2.68 ± 0.7**[a]
Nitrotyrosine (µmol/L)	0.013 ± 0.004
Malondialdehyde, MDA (µmol/L)	**2.48 ± 0.4**[a]
Serum Na^+ (mg/dL)	128.1 ± 6.1
Serum K^+ (mg/dL)	5.3 ± 1.1
Urea (mg/dL)	29.8 ± 2.4
Creatinine (mg/dL)	0.99 ± 0.2
Insulin (ng/mL)	**1.80 ± 0.42**[a]
Aldosterone (pg/mL)	119.5 ± 8.8
ACE activity (nmol/min/mL)	94.7 ± 8.2
Relative Target Organ Weight - g/100 g (Day 29)	
Heart	**0.35 ± 0.02**[a]
Spleen	0.16 ± 0.004
Right kidney	0.32 ± 0.01
Left kidney	**0.32 ± 0.01**[a]
Liver	**3.30 ± 0.13**[a]

Statistical analyses were performed using one-way ANOVA followed by Tukey's test. Data are presented as mean ± standard deviation. [a]p ≤ 0.05 indicates statistical significance compared to the naïve group. ACE: angiotensin-converting enzyme; ALT: alanine aminotransferase; AST: aspartate aminotransferase; cTNT: cardiac troponin T; DBP: diastolic blood pressure; EDV: end-diastolic volume; EF: ejection fraction; ESV: end-systolic volume; HDL: high-density lipoprotein; HR: heart rate; LDL: low-density lipoprotein; LVEDD: left ventricular end-diastolic diameter; LVESD: left ventricular end-systolic diameter; MAP: mean arterial pressure; MDA: malondialdehyde; SBP: systolic blood pressure; SV: stroke volume.

Legend to figure

Figure S1. Representative histological images of the liver, left kidney, left ventricle, and aortic arch from the naive, negative control, blank, HCTZ, α-amyrin-loaded nanocapsules, and αA (1 mg/kg; free α-amyrin) groups. H&E: hematoxylin and eosin stain.

Naïve group

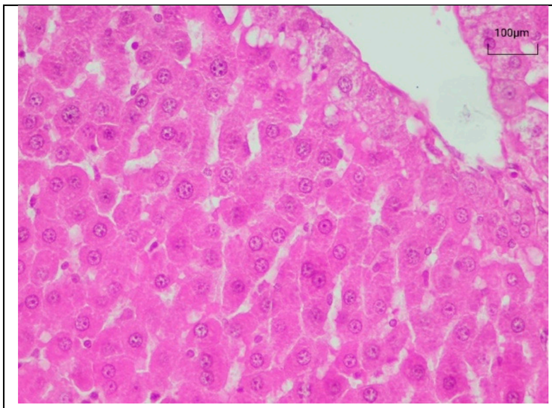	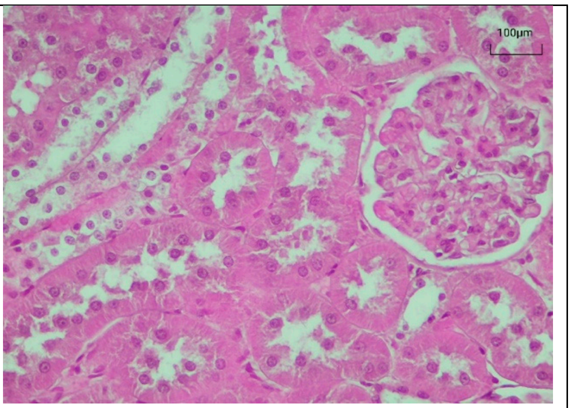
Liver, HE 40X	Left kidney, HE 40X
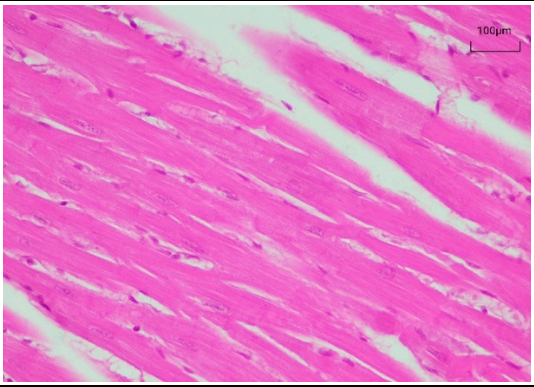	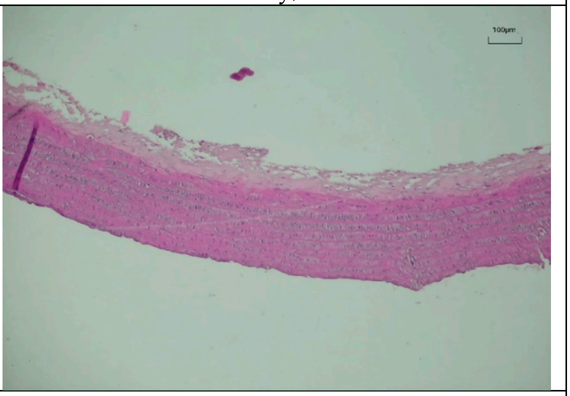
Left ventricle, HE 40X	Aortic arc, HE 10X

Negative control (NC) group

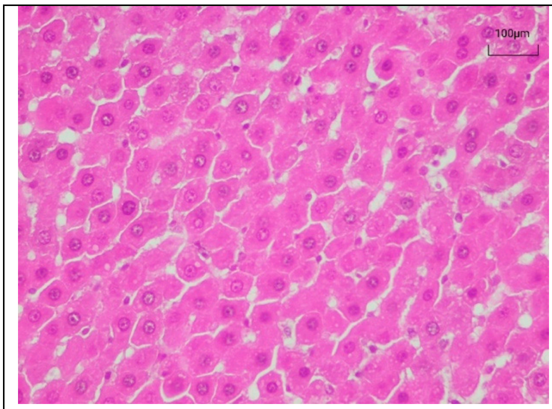

Liver, HE 40X

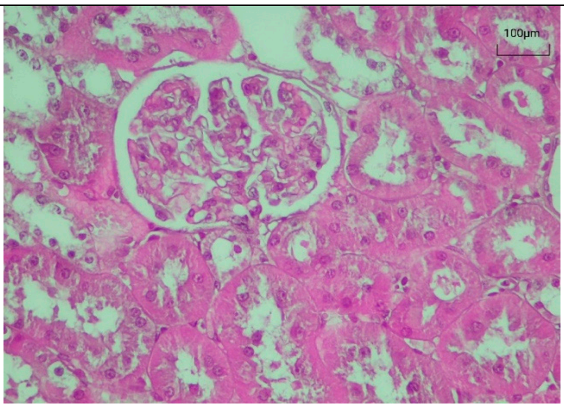

Left kidney, HE 40X

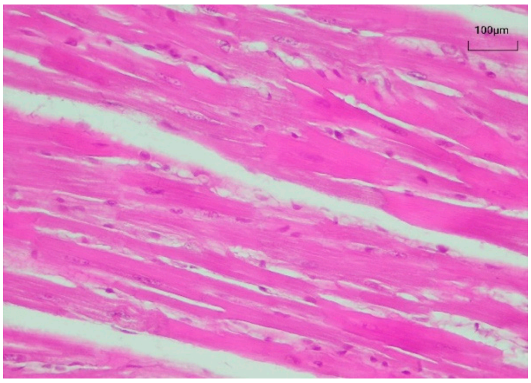

Left ventricle, HE 40X

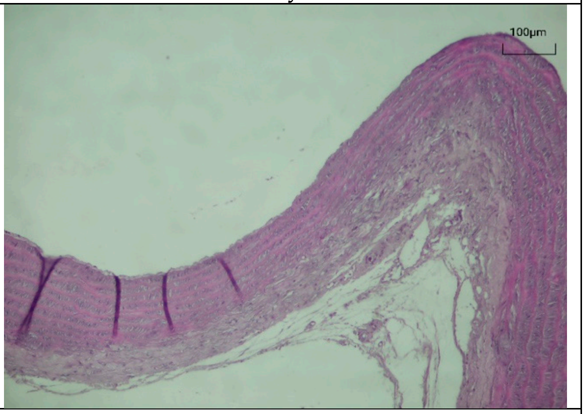

Aortic arc, HE 10X

Blank group (blank polymeric nanocapsule group)

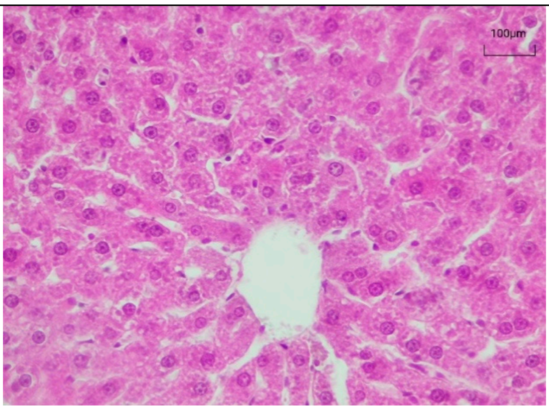

Liver, HE 40X

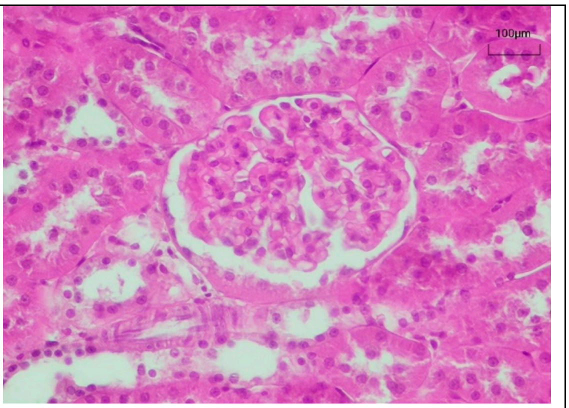

Left kidney, HE 40X

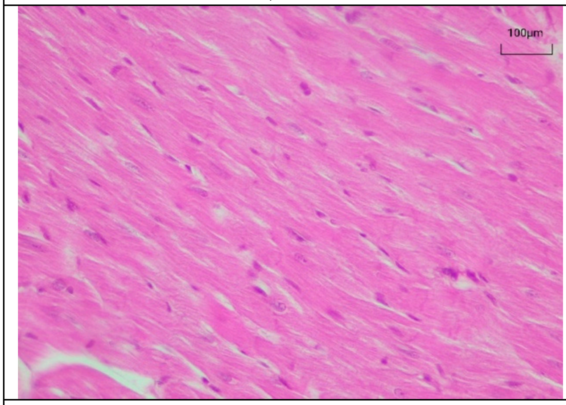

Left ventricle, HE 40X

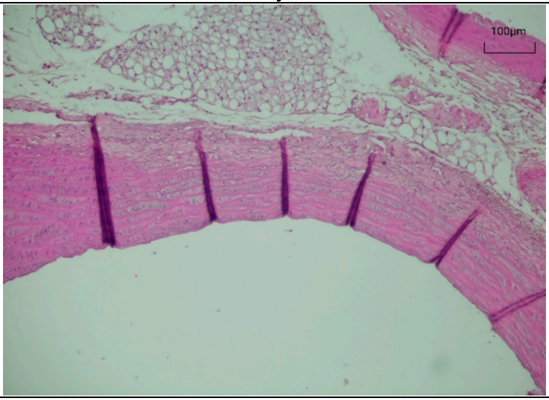

Aortic arc, HE 10X

HCTZ group (hydrochlorothiazide group)

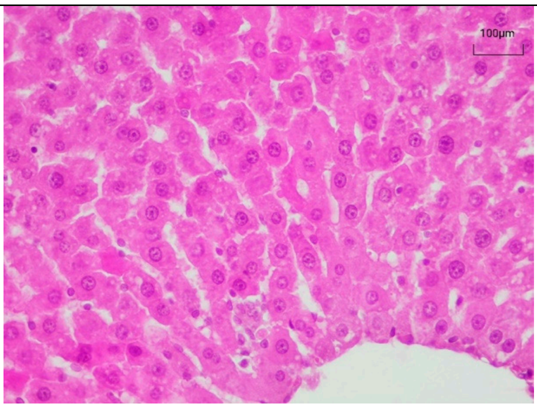

Liver, HE 40X

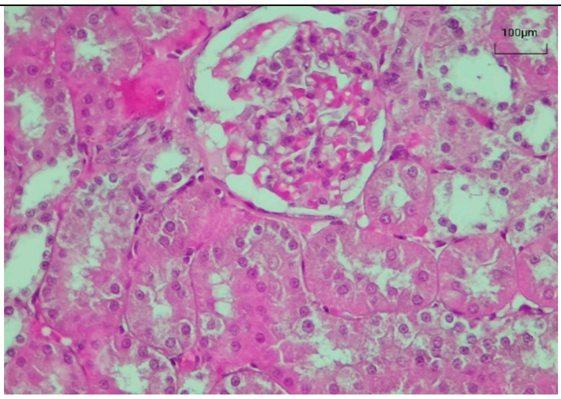

Left kidney, HE 40X

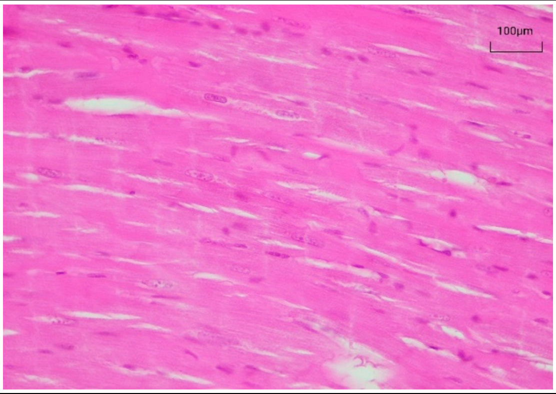

Left ventricle, HE 40X

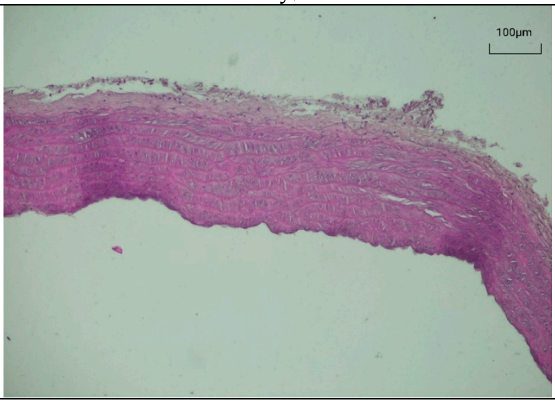

Aortic arc, HE 10X

NCαA group (1 mg/kg) (α-amyrin-loaded nanocapsules group)

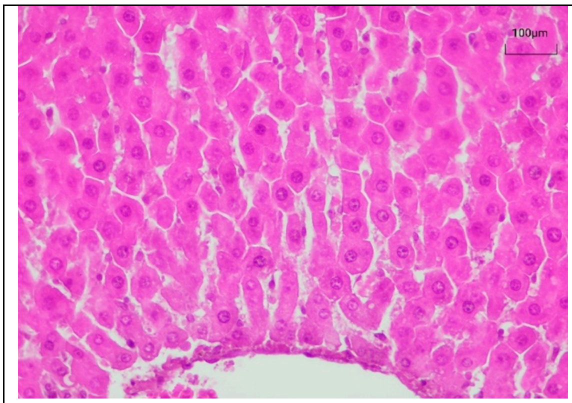

Liver, HE 40X

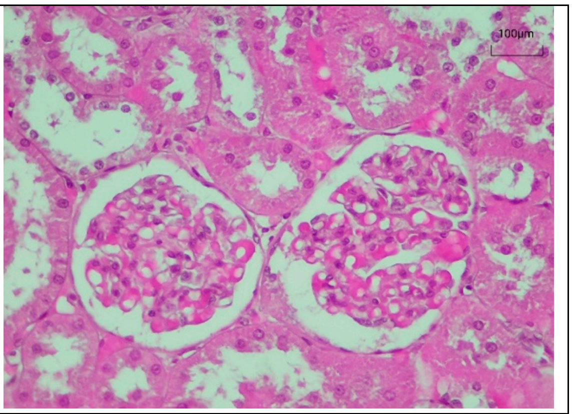

Left kidney, HE 40X

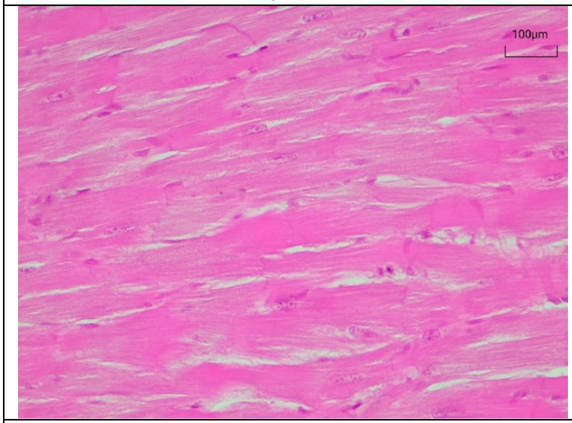

Left ventricle, HE 40X

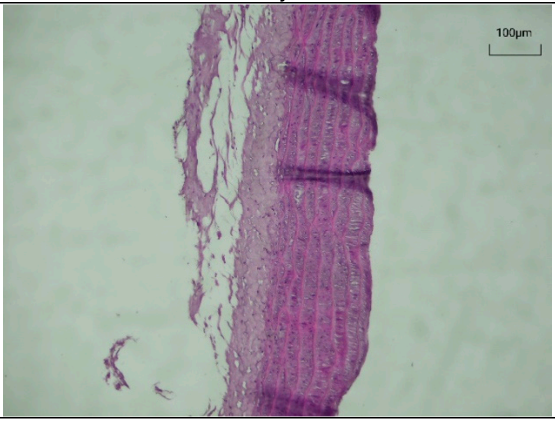

Aortic arc, HE 10X

αA group (1 mg/kg) (α-amyrin group)

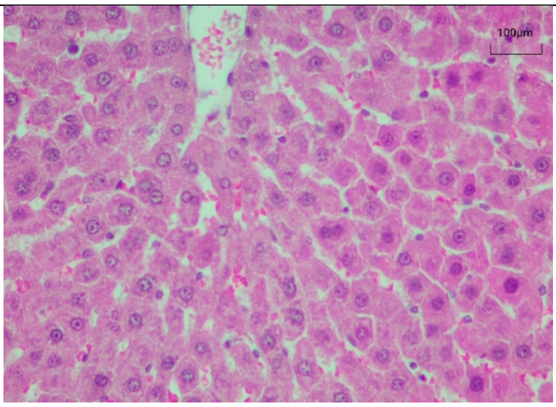	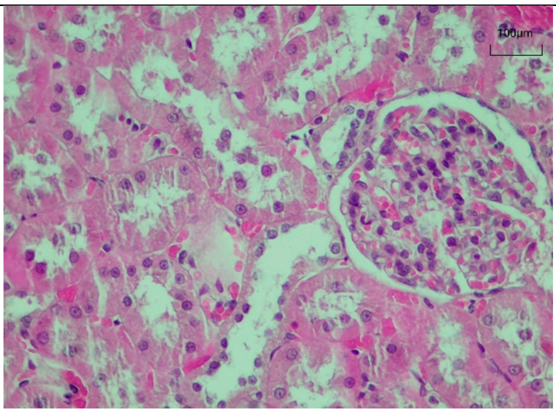
Liver, HE 40X	Left kidney, HE 40X
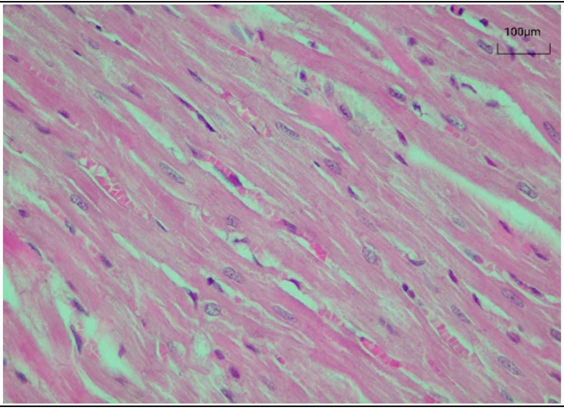	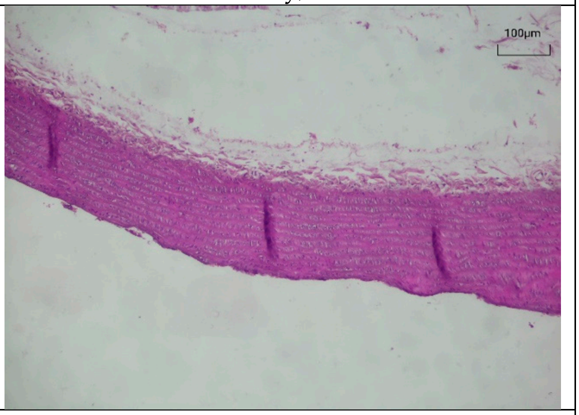
Left ventricle, HE 40X	Aortic arc, HE 10X
